# Supplementary material for: Intradermal-delivered DNA vaccine induces durable immunity mediating a reduction in viral load in a rhesus macaque SARS-CoV-2 challenge model
Source: Cell Rep Med. 2021 Sep 28;2(10):100420. doi: 10.1016/j.xcrm.2021.100420 (PMC8479327; doi:10.1016/j.xcrm.2021.100420)
Supplement: Document S1. Figures S1–S4 [file mmc1.pdf]

Cell Reports Medicine, Volume *2*

Supplemental information

Intradermal-delivered DNA vaccine induces durable immunity mediating a reduction in viral load in a rhesus macaque SARS-CoV-2 challenge model

Ami Patel, Jewell N. Walters, Emma L. Reuschel, Katherine Schultheis, Elizabeth Parzych, Ebony N. Gary, Igor Maricic, Mansi Purwar, Zeena Eblimit, Susanne N. Walker, Diana Guimet, Pratik Bhojnagarwala, Opeyemi S. Adeniji, Arthur Doan, Ziyang Xu, Dustin Elwood, Sophia M. Reeder, Laurent Pessaint, Kevin Y. Kim, Anthony Cook, Neethu Chokkalingam, Brad Finneyfrock, Edgar Tello-Ruiz, Alan Dodson, Jihae Choi, Alison Generotti, John Harrison, Nicholas J. Tursi, Viviane M. Andrade, Yaya Dia, Faraz I. Zaidi, Hanne Andersen, Mohamed Abdel-Mohsen, Mark G. Lewis, Kar Muthumani, J. Joseph Kim, Daniel W. Kulp, Laurent M. Humeau, Stephanie J. Ramos, Trevor R.F. Smith, David B. Weiner, and Kate E. Broderick

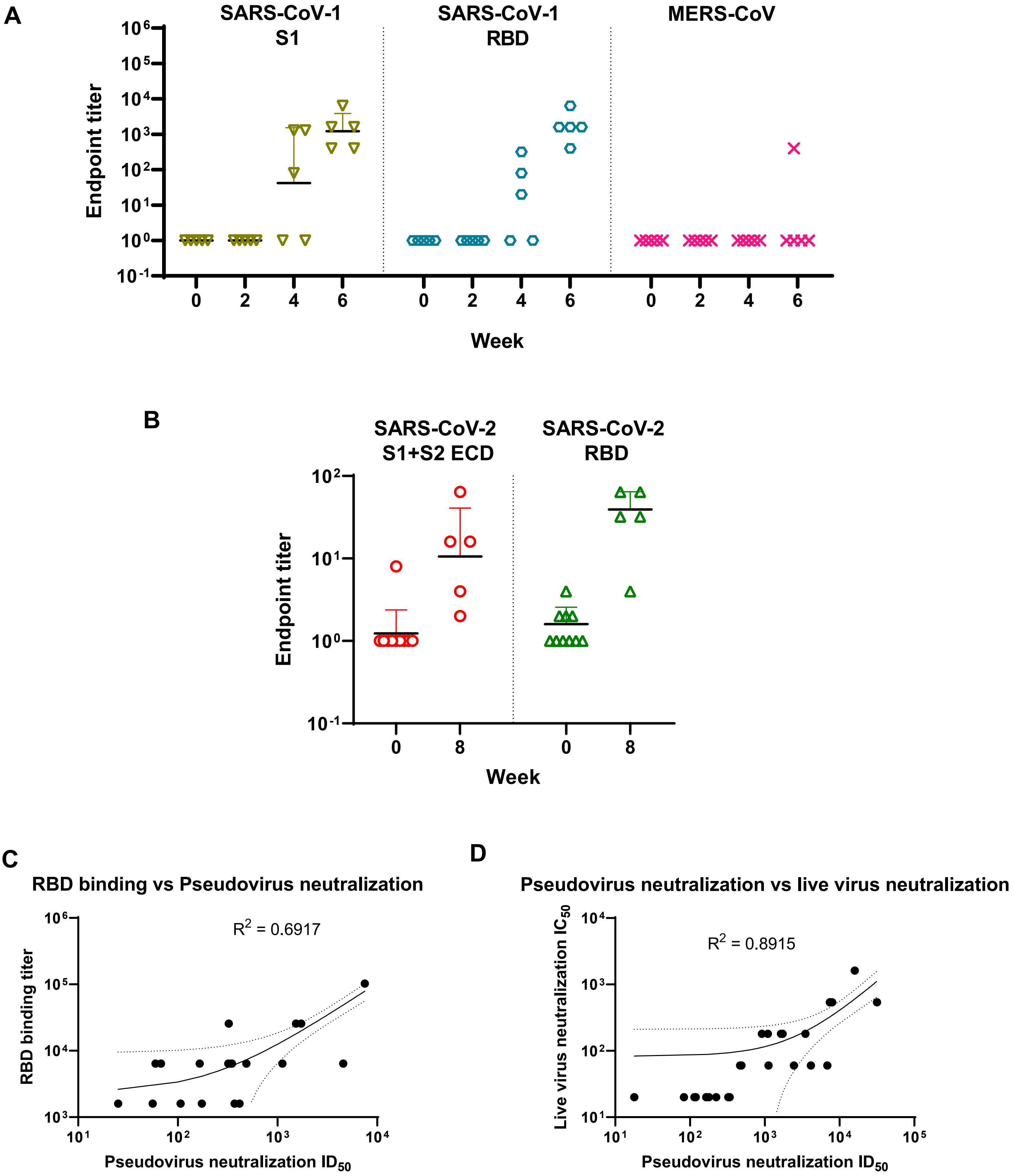

Figure S1. Immune Responses in INO-4800 vaccinated rhesus macaques, Related to Figure 1 **A)** Serum IgG cross-reactivity to SARS-CoV and MERS-CoV spike protein. IgG binding was measured in sera from INO-4800 vaccinated rhesus macaques to SARS-CoV S1 and MERS-CoV S1 protein antigen. **B)** Bronchoalveolar lavage (BAL) IgG reactive to SARS-CoV-2 S protein antigens. BAL samples collected from vaccinated animals were assessed for SARS-CoV-2 reactive IgG binding to the full-length SARS-CoV-2 spike protein and the RBD domain. **C and D)** Simple linear regression analysis comparing total IgG antibodies against (**C**) the RBD and (**D**) neutralizing antibodies raised in DNA-vaccinated macaques

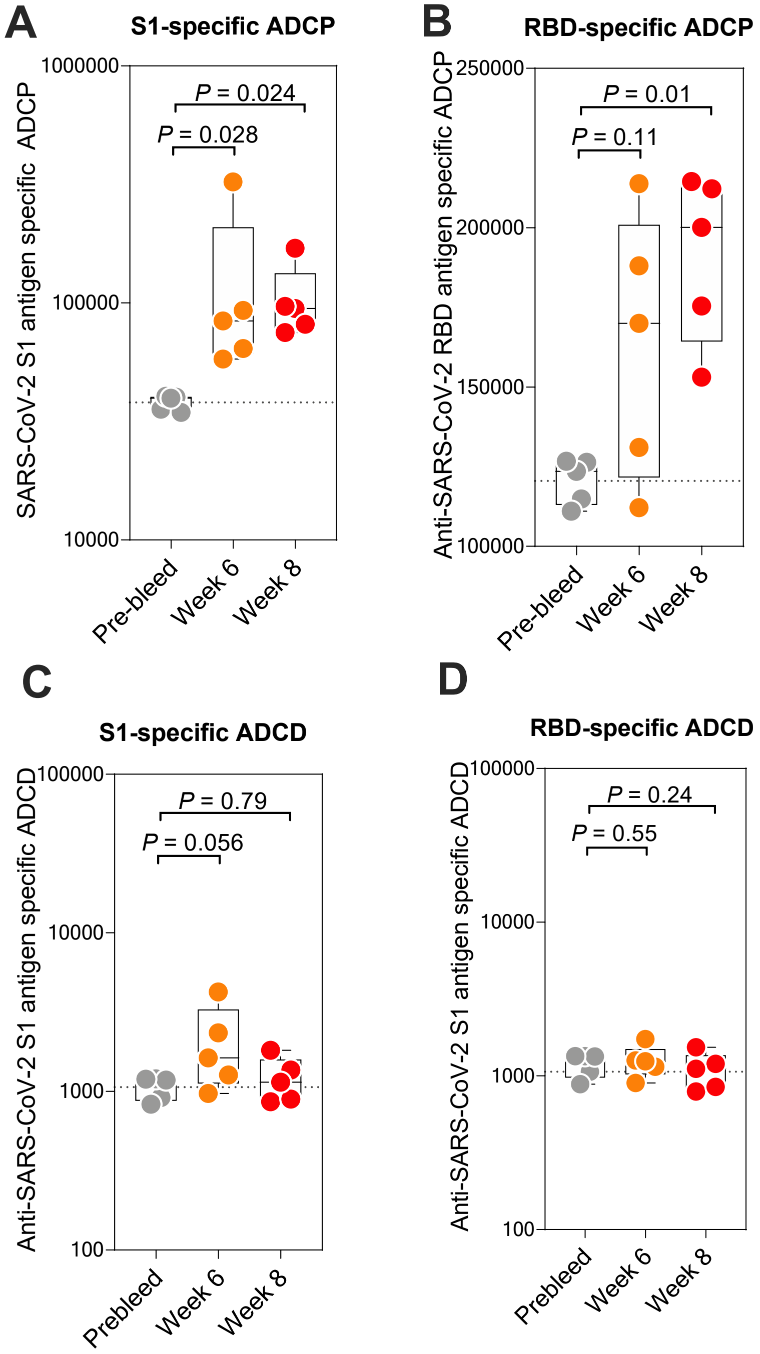

Figure S2. Antibody-dependent cellular phagocytosis (ADCP) and antibody-dependent complement deposition (ADCD) activity of NHP sera at peak immune response time points post-vaccination, Related to Figure 1. To measure ADCP activity, sera from immunized macaques were combined with SARS-CoV-2 A) S1 or B) RBD-conjugated fluorescent beads, followed by overnight incubation with THP-1 cells. The phagoscore was determined by calculating the percentage of fluorescent / bead+ cells and the median fluorescence intensity of the THP-1 cells. To measure ADCD activity, ACE2-CHO cells were pulsed with biotinylated SARS-CoV-2 C) S1 and D) RBD proteins and subsequently combined with NHP sera. Freshly diluted guinea pig complement was added to the cells and following incubation, complement deposition was detected with a goat anti-guinea pig C3-FITC conjugated antibody and analyzed by flow cytometry. ADCD is reported as MFI of FITC+ cells.

A

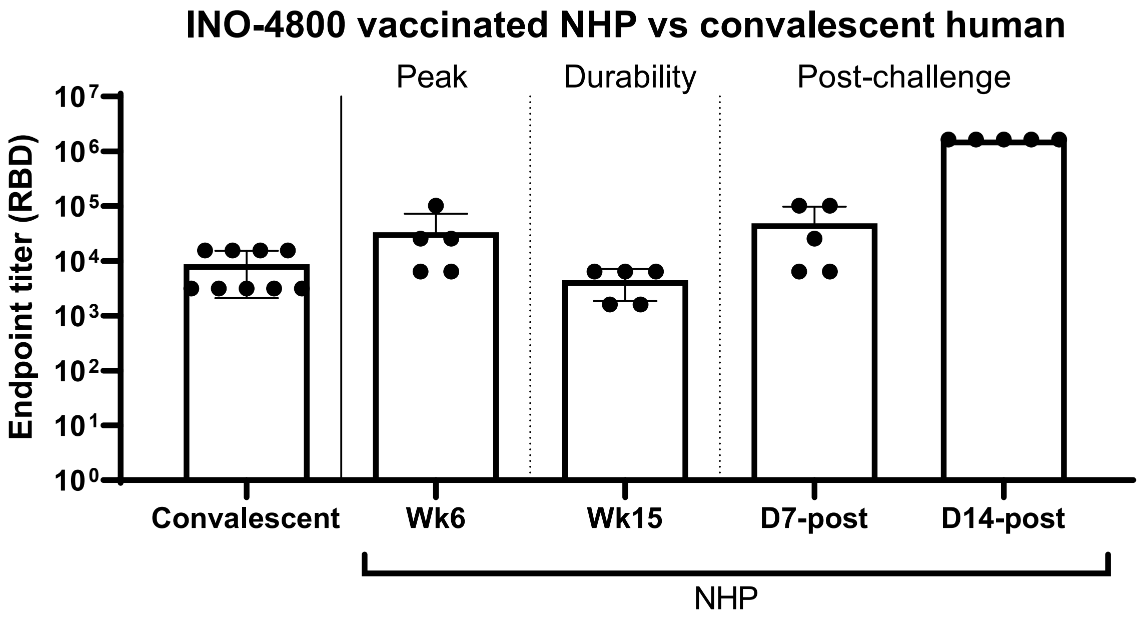

B

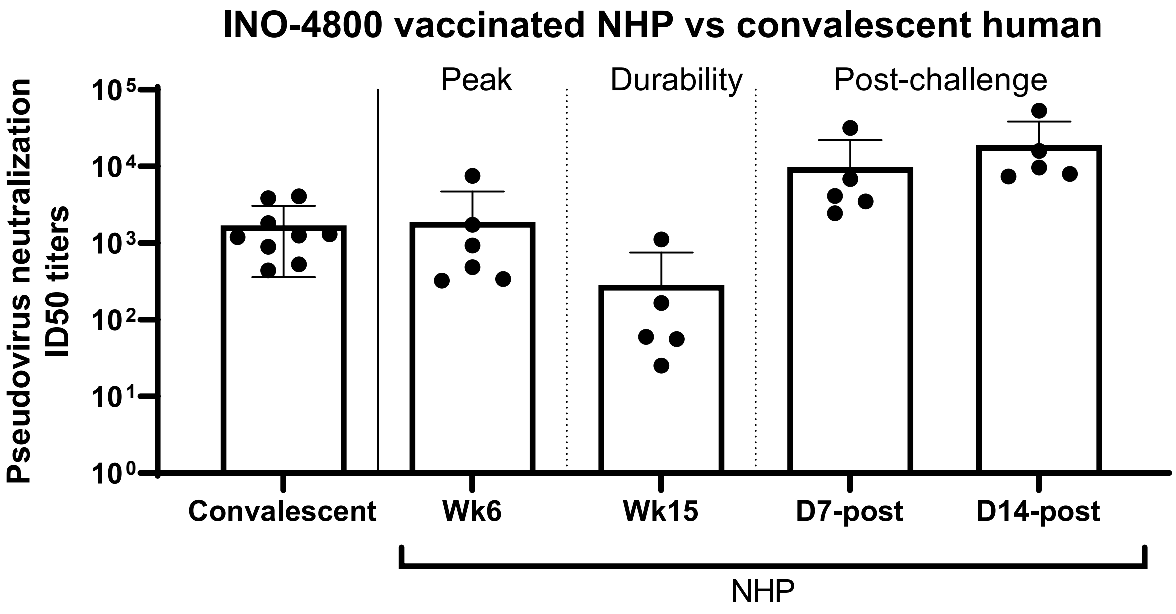

Figure S3. Comparison of NHP endpoint binding antibody and pseudoneutralization titers with SARS-CoV-2 convalescent human donors, Related to Figures 1 and 3. (**A**) ELISA endpoint titer comparison (RBD) and (**B**) Pseudoneutralization assay (D614) comparing INO-4800 vaccinated NHP peak, durability, and post-challenge pseudoneutralization ID50 titers with SARS-CoV-2 convalescent human sera titers.

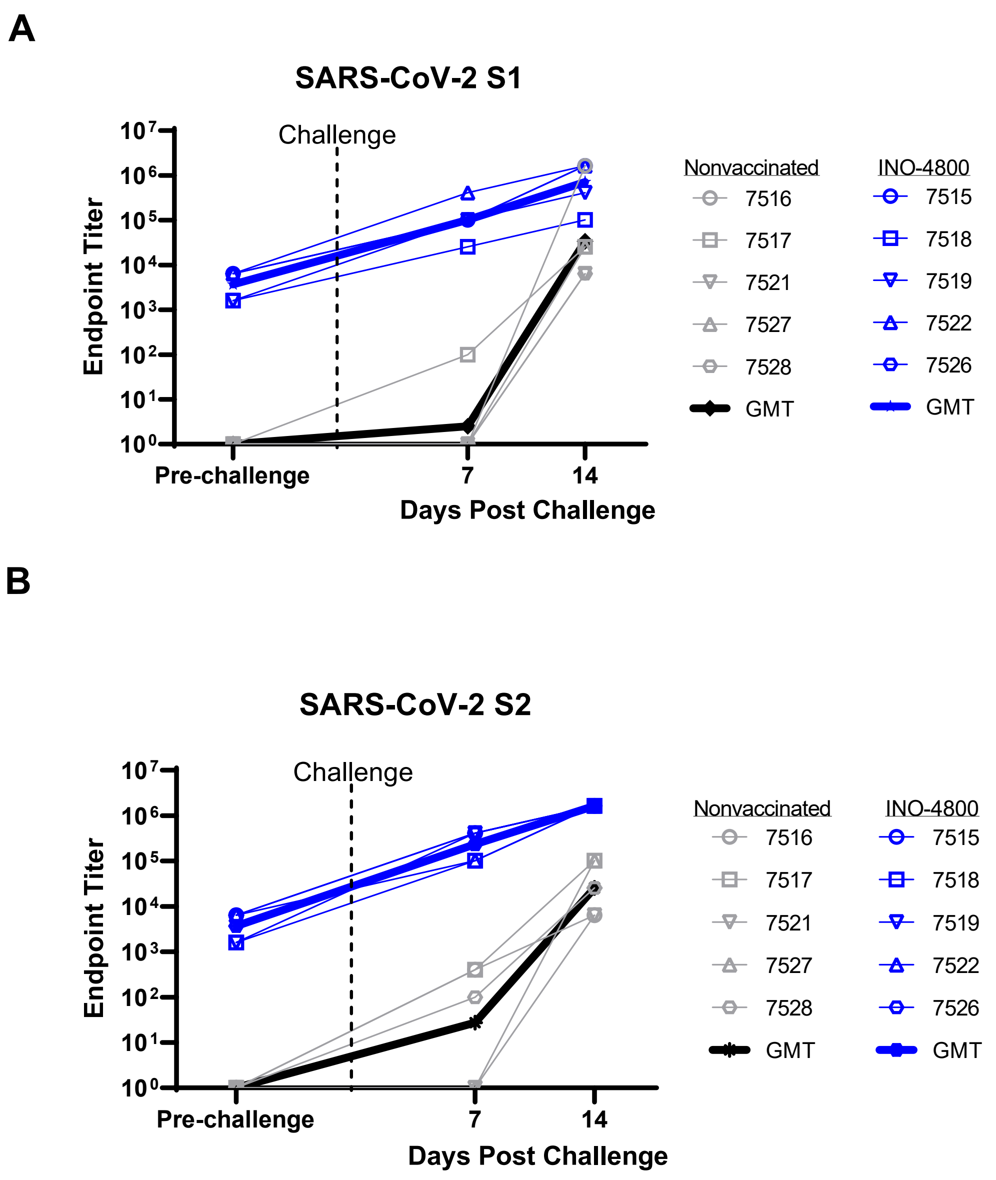

Figure S4. **Recall of humoral immune responses after viral challenge, Related to Figure 3.** (**A**) SARS-CoV-2 S1 protein and (**B**) SARS-CoV-2 S2 protein antigen binding of IgG in diluted NHP sera collected prior to challenge, during challenge and post challenge.
