# Supplementary material for: Intradermal-delivered DNA vaccine induces durable immunity mediating a reduction in viral load in a rhesus macaque SARS-CoV-2 challenge model
Source: Cell Rep Med. 2021 Sep 28;2(10):100420. doi: 10.1016/j.xcrm.2021.100420 (PMC8479327; doi:10.1016/j.xcrm.2021.100420)
Supplement: Document S2. Article plus supplemental information [file mmc2.pdf]

Cell Reports
Medicine

Article

Intradermal-delivered DNA vaccine induces durable immunity mediating a reduction in viral load in a rhesus macaque SARS-CoV-2 challenge model

Graphical abstract

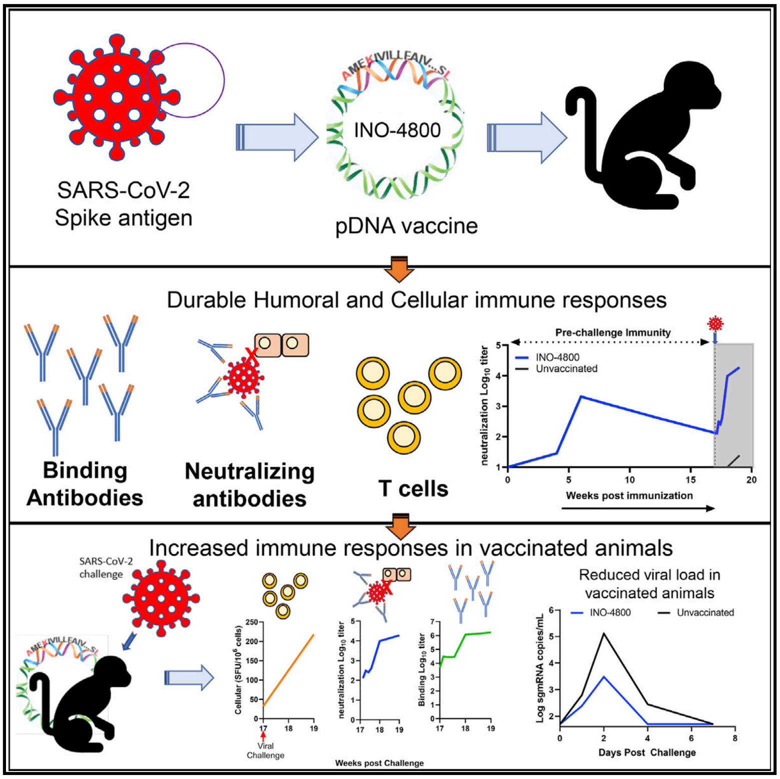

Highlights

- INO-4800 induces cellular and humoral immune responses in macaques
- Vaccinated animals respond rapidly to SARS-CoV-2 exposure 17 weeks post-immunization
- This study employs a clinically relevant intradermal vaccine dose and regimen

Authors

Ami Patel, Jewell N. Walters, Emma L. Reuschel, ..., Trevor R.F. Smith, David B. Weiner, Kate E. Broderick

Correspondence

kate.broderick@inovio.com (K.E.B.), dweiner@wistar.org (D.B.W.)

In brief

Patel et al. demonstrate that immunization with a DNA vaccine encoding the SARS-CoV-2 spike antigen, INO-4800, induces durable immune responses in rhesus macaques and is associated with reduced viral loads after challenge.

Patel et al., 2021, Cell Reports Medicine 2, 100420
October 19, 2021 © 2021 The Author(s).
https://doi.org/10.1016/j.xcrm.2021.100420

CellPress

Cell Reports Medicine

CellPress
OPEN ACCESS

Article

Intradermal-delivered DNA vaccine induces durable immunity mediating a reduction in viral load in a rhesus macaque SARS-CoV-2 challenge model

Ami Patel,[1,4] Jewell N. Walters,[2,4] Emma L. Reuschel,[1,4] Katherine Schultheis,[2,4] Elizabeth Parzych,[1,4] Ebony N. Gary,[1,4] Igor Maricic,[2] Mansi Purwar,[1] Zeena Eblimit,[2] Susanne N. Walker,[1] Diana Guimet,[2] Pratik Bhojnagarwala,[1] Opeyemi S. Adeniji,[1] Arthur Doan,[2] Ziyang Xu,[1] Dustin Elwood,[2] Sophia M. Reeder,[1] Laurent Pessaint,[3] Kevin Y. Kim,[1] Anthony Cook,[3] Neethu Chokkalingam,[1] Brad Finneyfrock,[3] Edgar Tello-Ruiz,[1] Alan Dodson,[3] Jihae Choi,[1] Alison Generotti,[2] John Harrison,[3] Nicholas J. Tursi,[1] Viviane M. Andrade,[2] Yaya Dia,[1] Faraz I. Zaidi,[1] Hanne Andersen,[3] Mohamed Abdel-Mohsen,[1] Mark G. Lewis,[3] Kar Muthumani,[1] J. Joseph Kim,[2] Daniel W. Kulp,[1] Laurent M. Humeau,[2] Stephanie J. Ramos,[2] Trevor R.F. Smith,[2] David B. Weiner,[1,*] and Kate E. Broderick[2,5,*]

[1]Vaccine and Immunotherapy Center, The Wistar Institute, Philadelphia, PA 19104, USA
[2]Inovio Pharmaceuticals, Plymouth Meeting, PA 19462, USA
[3]Bioqual, Rockville, MD 20852, USA
[4]These authors contributed equally
[5]Lead contact
*Correspondence: kate.broderick@inovio.com (K.E.B.), dweiner@wistar.org (D.B.W.)
https://doi.org/10.1016/j.xcrm.2021.100420

SUMMARY

Coronavirus disease 2019 (COVID-19), caused by the SARS-CoV-2 virus, has had a dramatic global impact on public health and social and economic infrastructures. Here, we assess the immunogenicity and anamnestic protective efficacy in rhesus macaques of an intradermal (i.d.)-delivered SARS-CoV-2 spike DNA vaccine, INO-4800, currently being evaluated in clinical trials. Vaccination with INO-4800 induced T cell responses and induced spike antigen and RBD binding antibodies with ADCP and ADCD activity. Sera from the animals neutralized both the D614 and G614 SARS-CoV-2 pseudotype viruses. Several months after vaccination, animals were challenged with SARS-CoV-2 resulting in rapid recall of anti-SARS-CoV-2 spike protein T cell and neutralizing antibody responses. These responses were associated with lower viral loads in the lung. These studies support the immune impact of INO-4800 for inducing both humoral and cellular arms of the adaptive immune system, which are likely important for providing durable protection against COVID-19 disease.

INTRODUCTION

COVID-19 was declared a global pandemic on March 11, 2020 by the World Health Organization. There are >160 million confirmed cases worldwide with the total number of deaths estimated to be >3,466,670 (May 24, 2021, https://www.gisaid.org/). COVID-19 presents as a respiratory illness, with mild-to-moderate symptoms in many cases (~80%).[1,2] These symptoms include headache, cough, fever, fatigue, difficulty breathing, and possible loss of taste and smell. The factors involved with progression to severe COVID-19 disease in 20% of cases are unclear; yet severe disease is characterized by development of a hyperinflammatory response, followed by development of acute respiratory distress syndrome (ARDS), potentially leading to mechanical ventilation, kidney failure, and death.[2,3] The rapid development of vaccine countermeasures remains a high priority for this infection, with multiple candidates having entered the clinic in record time.[4] Several vaccines have now been approved through emergency-use authorization (EUA).[5–7] However, the number of vaccine doses available only covers a small percentage of the global population, and more vaccines are urgently needed.

SARS-CoV-2 is a positive-sense single-stranded RNA virus belonging to the family *Coronaviridae*, genus *Betacoronavirus*, the same family as severe acute respiratory syndrome coronavirus (SARS-CoV) and Middle East respiratory syndrome coronavirus (MERS-CoV). SARS-CoV-2 is homologous to SARS-CoV, sharing around 70%–80% of its genome.[8] Structural components of SARS-CoV-2 include the envelope (E) protein, membrane (M) protein, nucleocapsid (N) protein, and surface spike (S) protein, which is a major immunogenic target for humoral and cellular immune responses. Virus entry into host cells is mediated through S protein receptor binding domain (RBD) interaction with the host cell receptor angiotensin converting enzyme 2 (ACE2) and priming by the serine protease TMPRSS2.[9] Recent preprints and published studies also implicate S1 binding to neuropilin 1 (NLP-1)[10,11] and have identified neutralizing epitopes outside RBD.[12]

SARS-CoV-2 is the third coronavirus outbreak this century. Prior work with the related coronaviruses, SARS-CoV and

© 2021 The Author(s).
This is an open access article under the CC BY license (http://creativecommons.org/licenses/by/4.0/).

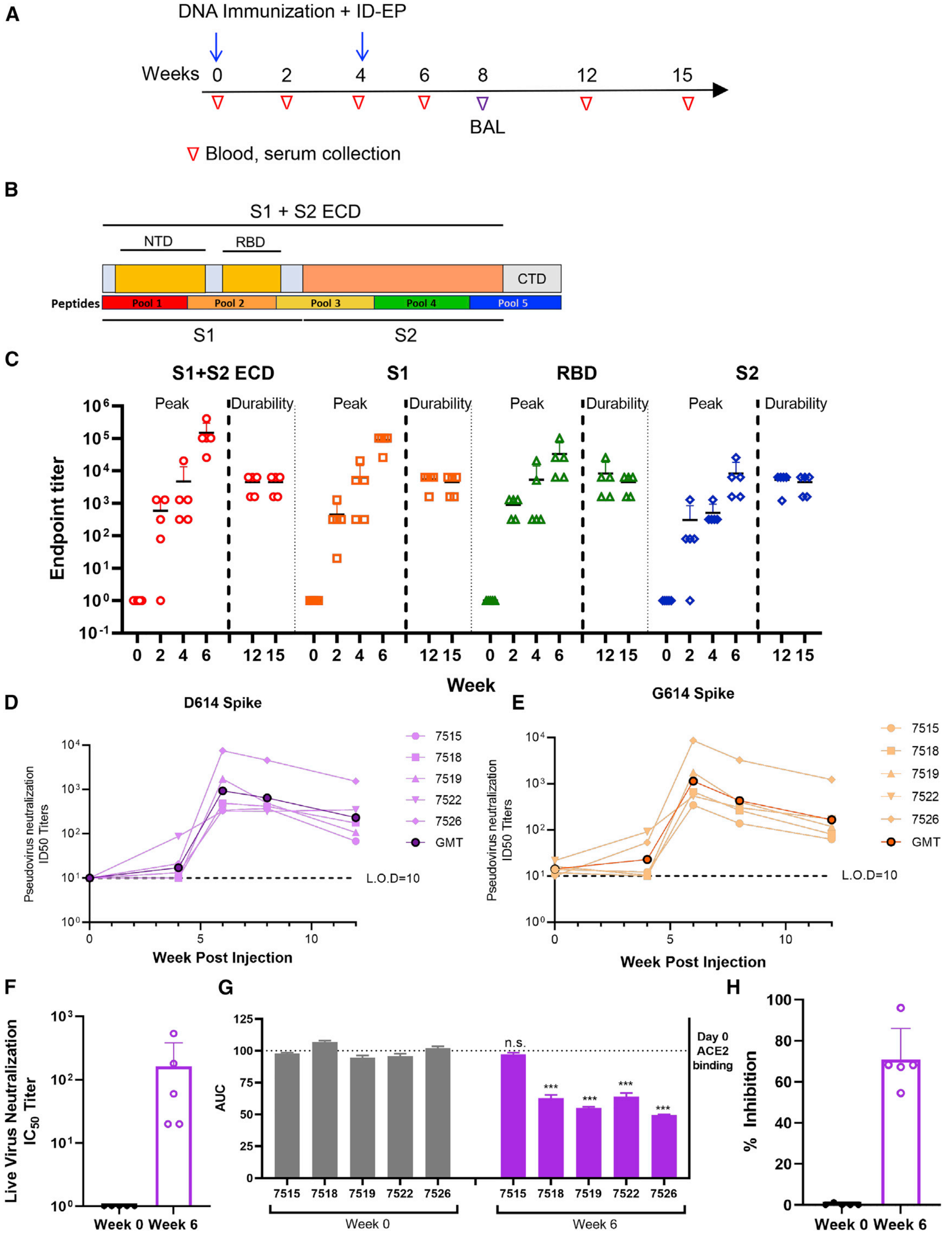

Figure 1. Humoral immune responses in rhesus macaques

(A) The study outline showing the vaccination regimen and sample collection time points.

(B) Schematic of SARS-CoV-2 spike protein.

(C) SARS-CoV-2 S1+S2 ECD, S1, RBD, and S2 protein antigen binding of IgG in serially diluted NHP sera. The data represent the mean endpoint titers for each individual NHP.

(D and E) Pseudovirus neutralization assay using NHP sera, showing the presence of SARS-CoV-2-specific neutralizing antibodies against the D614 (D) and G614 (E) variants of SARS-CoV-2.

(F) Live virus neutralization using NHP serum. Isolate USA-WA1/2020.

(legend continued on next page)

MERS-CoV, delineated that the Spike protein of these viruses was an important target for development of neutralizing antibodies and vaccine targeted immunity in animal models of viral challenge (reviewed in previous studies[4,13–17]).

Many studies have described initial data for vaccines developed from inactivated viruses,[18] recombinant adenoviral vectors that express the spike antigen,[19,20] mRNA vaccine,[21,22] protein nanoparticles,[23] or intramuscular (IM) delivered DNA vaccine candidates[24] in non-human primates (NHPs). These studies focused on disease protection in acute models with challenge weeks after the final vaccination, demonstrating the ability of vaccination to lower viral load in the lungs, with less impact on viral loads in the nose.[19,25] Here, we report on the impact of an ID-delivered SARS-CoV-2 DNA vaccine in a nonhuman primate model where the animals were challenged 3 months post-vaccination.

Recent studies support an important role for both cellular and humoral immunity in protection and recovery from COVID-19 disease.[26–28] T cell-mediated immunity has been shown to be important for other beta-coronaviruses, providing both direct protection and help in the generation of effective humoral responses.[29,30] Several SARS-CoV-2 studies have now reported that up to 40% of persons can exhibit mild disease, which may be associated with T cell immunity[31] or humoral responses[8] and have indicated the levels of SARS-CoV-2 antibodies wane rapidly in convalescent subjects.[32] This echoes older studies that found that neutralizing antibody levels and memory B cell responses have not been sustained in SARS survivors, while responsive T cell populations have proved more durable.[33–35] Furthermore, a SARS-CoV-2 challenge study in non-human primates (NHPs) indicated that CD8 T cells had a major contribution to the reduction in viral loads for animals with low anti-SARS-CoV-2 IgG levels.[36]

We have described the immunogenicity and efficacy of a SARS-CoV-2 DNA vaccine (INO-4800), encoding a synthetic spike immunogen in small animal models.[37] This vaccine candidate induced neutralizing and ACE2-blocking antibodies, as well as T cell responses in mice and guinea pigs. Here, we assess vaccine-induced T and B cell responses during the acute expansion phase in rhesus macaques, as well as the impact infection in an NHP viral installation challenge model. Additionally, we studied the effect of the vaccines on the emergence of the dominant SARS-CoV2 variant G614, which now comprises more than 80% of circulating global viral strains and is reportedly associated with increased infectivity and spread.[38] We report vaccine-induced humoral and cellular immunity, including neutralizing antibody responses against both the SARS-CoV-2 D614 virus as well as the G614 strain. Upon SARS-CoV-2 challenge, more than 3 months following the final dose, vaccinated macaques exhibited a rapid recall response against multiple regions of the S protein. This anamnestic response was characterized by expansion of neutralizing antibody responses, as well as the rapid expansion of T cell responses. These immune responses induced by INO-4800 were associated with a reduction of viral load in the lower respiratory tract.

RESULTS

Induction of humoral and cellular immune responses in INO-4800-immunized non-human primates

NHPs are a valuable model in the development of COVID-19 vaccines and therapeutics as they can be infected with wild-type SARS-CoV-2 and present with early infection that mimics aspects of human disease.[18,39] Rhesus macaques (n = 5) received two immunizations of INO-4800 (1 mg), at weeks 0 and 4 (Figure 1A). Naive control animals (n = 5) did not receive vaccine. Humoral and cellular immune responses were monitored for 15 weeks (~4 months) following prime immunization for antigen-specific T cell responses. All animals seroconverted following a single INO-4800 immunization, with serum immunoglobulin G (IgG) titers detected against the full-length S1+S2 extracellular domain (ECD), S1, S2, and RBD regions of the SARS-CoV-2 S protein (Figures 1B and 1C). Cross-reactive antibodies were also detected against SARS-CoV S1 protein and RBD but not MERS-CoV (Figure S1A). SARS-CoV-2 spike antigen-reactive IgG against the S1 + S2 ECD and RBD were detected in bronchoalveolar lavage (BAL) washes at week 8, 4 weeks following the 2nd immunization dose (Figure S1B).

SARS-CoV-2 pseudovirus-neutralizing antibodies were measured in the serum of animals between study weeks 0 and 12 (Figure 1D). During the course of the COVID-19 pandemic, a D614G SARS-CoV-2 spike variant has emerged that has potentially greater infectivity and now accounts for >80% of new isolates. We evaluated neutralization against this new variant using a modified pseudovirus developed to express the G614 spike protein (Figure 1E). Similar neutralization ID50 titers were observed against both D614 and G614 spikes, supporting induction of functional antibody responses by the vaccine against both SARS-CoV-2 virus variants. A live virus neutralization assay was performed on serum samples collected at week 0 and 6 (Figure 1F). At week 6, neutralization of viral isolate USA-WA1/2020 was detected in all animals with IC_{50}s ranging from 20 to 540. A simple linear regression analyses comparing RBD binding titers versus pseudovirus neutralization titers and live virus versus pseudovirus neutralization titers showed positive correlations of R^2 = 0.6917 and R^2 = 0.8915, respectively (Figures S1C and S1D).

To investigate specific receptor-blocking neutralizing antibody activity, we assessed the capacity to occlude ACE2 binding of SARS-CoV-2 spike antigen by vaccine-induced sera. We recently developed a receptor-blocking assay and demonstrated that it correlates with pseudovirus neutralization titers.[40] In this assay, anti-spike antibodies are bound to SARS-CoV-2 spike protein in an ELISA plate followed by addition of a soluble version of the ACE2 receptor. Addition of sera-containing antibodies, which can obstruct the receptor binding site on the spike

(G and H) Serum collected at week 6 from INO-4800-vaccinated NHPs inhibited ACE2 binding to SARS-CoV-2 Spike protein. A plate-based ACE2 competition assay (G) and a flow cytometry-based ACE2 competition assay (H) and showing inhibition of ACE2 binding to Spike protein by NHP sera. Bars represent the mean ± SD. ***$p < 0.001$.
See also Figures S1–S3.

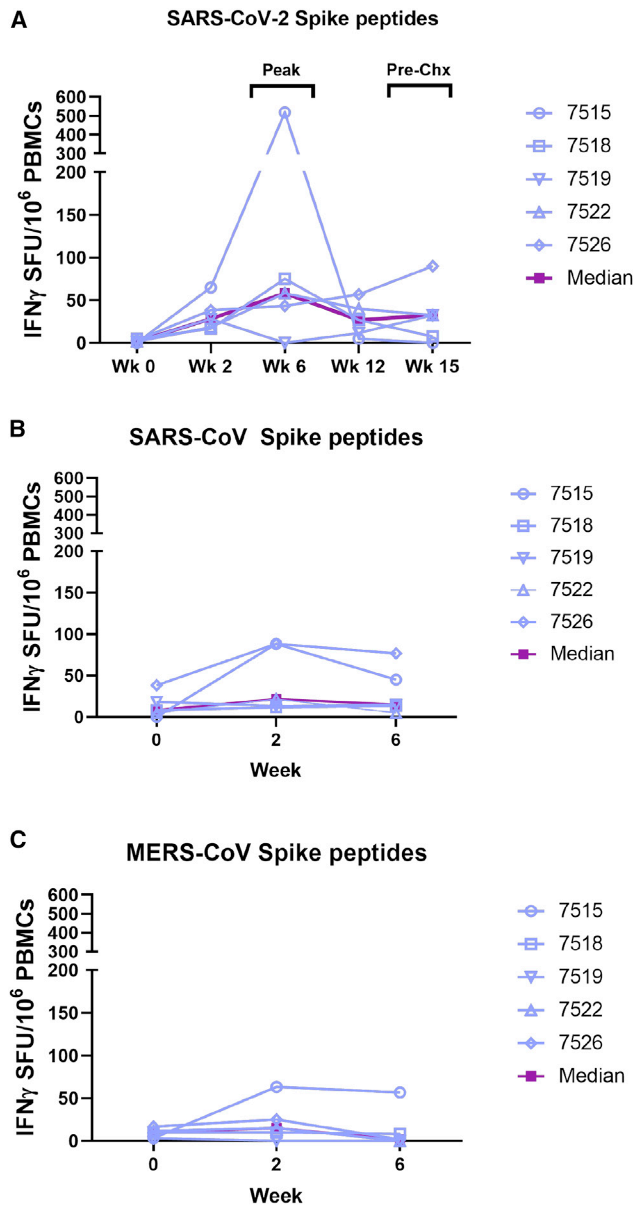

Figure 2. Cellular immune responses in rhesus macaques

(A) T cell responses were measured by IFN-γ ELISpot in PBMCs stimulated for 20 h with overlapping peptide pools spanning the SARS-CoV-2 Spike protein.
(B and C) Cross-reactivity to SARS-CoV and MERS-CoV spike protein were analyzed by IFN-γ ELISpot after 20 h stimulation with overlapping peptide pools spanning the SARS-CoV-1 spike protein (B) and MERS-CoV spike protein (C). Individual animal responses are depicted by open symbols and filled symbols represent median values.

protein, will lead to readings of lower area under the curve (AUC) values. We observed that sera from 80% of immunized NHPs had reduced AUCs indicating the presence of antibodies that can block the SARS-CoV-2 spike protein from interacting with ACE2 (Figure 1G). An independent flow-cytometry-based assay was also performed to further study the Spike-ACE2 interaction. ACE2-expressing 293T cells were co-incubated with Spike with or without the presence of sera. Spike binding to ACE2 was detected by flow cytometry. In this assay, 100% of macaques generated vaccine-induced antibodies that inhibited the Spike-ACE2 interaction, with a range of 53%–96% inhibition (Figure 1H). It should be noted this assay was conducted with a 1:27 dilution of sera, but subsequent dilutions of the sera yielded similar results with ~50% inhibition.

Antibody-dependent cellular phagocytosis (ADCP) and antibody-dependent complement deposition (ADCD) assays were performed using sera collected at baseline, week 6, and week 8 post-immunization to assess the ability of sera from immunized NHPs to engage effector functions. ADCP activity against the SARS-CoV-2 S1 (Figure S2A) and RBD (Figure S2B) proteins was significantly increased above baseline for immunized NHPs at week 8 ($p = 0.024$ and $p = 0.01$, respectively). There was also an increased trend of ADCD activity against SARS-CoV-2 S1 protein for immunized NHPs at week 6 ($p = 0.056$) (Figure S2C). No differences were observed for ADCD activity against the RBD (Figure S2D).

Total serum antibody and pseudovirus neutralization titers from immunized NHPs were compared with titers detected from 9 human SARS-CoV-2 convalescent sera donors (3, asymptomatic, 3 mild, and 3 moderate infections, sera collected between 30 and 80 days following positive COVID-19 PCR diagnosis). Overall, ELISA endpoint binding titers against RBD and pseudoneutralization titers at the peak immune response in NHPs (6 weeks post-immunization) were similar to those observed in convalescent donors (Figures S3A and S3B).

INO-4800 immunization induced SARS-CoV-2 S antigen reactive T cell responses against all 5 peptide pools with T cells responses peaking at week 6 (0–518 SFU/million cells with 4/5 NHPs showing positive responses) (Figure 2A). Cross-reactive T cells responses were also detected against the SARS-CoV spike protein in all NHPs and peaked after the first vaccination (12–88 SFU/million cells), which could likely be attributed to the high degree of sequence homology between SAR-CoV and SARS-CoV-2 (Figure 2B). These responses start to decline by week 6 (5–76 SFU/million cells), possibly suggesting competitive expansion of SARS-CoV-2-specific T cells. We did not observe cross-reactivity to MERS-CoV spike peptides, which is consistent with the lower sequence homology present between SARS-CoV-2 and MERS-CoV (Figure 2C).

Vaccine induced immune recall responses upon SARS-CoV-2 challenge in non-human primates

INO-4800-vaccinated macaques and unvaccinated controls were challenged with SARS-CoV-2 at study week 17 (Figure 3A). NHPs received a challenge dose of 1.1×10^4 PFU of SARS-CoV-2 Isolate USA-WA1/2020 by intranasal and intratracheal inoculation, as previously described.[20,25,39] By day 7, all vaccinated animals had an increase in antibody titers against the SARS-CoV-2 S1+S2 ECD, S1, RBD, and S2 proteins (Figures 3B, 3C, and S4). Seven days post-challenge, robust geometric mean endpoint titers ranging from 409,600 to 1,638,400 for the S1+S2 ECD and 6,400 to 102,400 for RBD were observed in immunized animals, compared with the naive group which displayed seroconversion of only 1/5 animals (Figures 3B and 3C). Antibody titers continued to increase against SARS-CoV-2 Spike

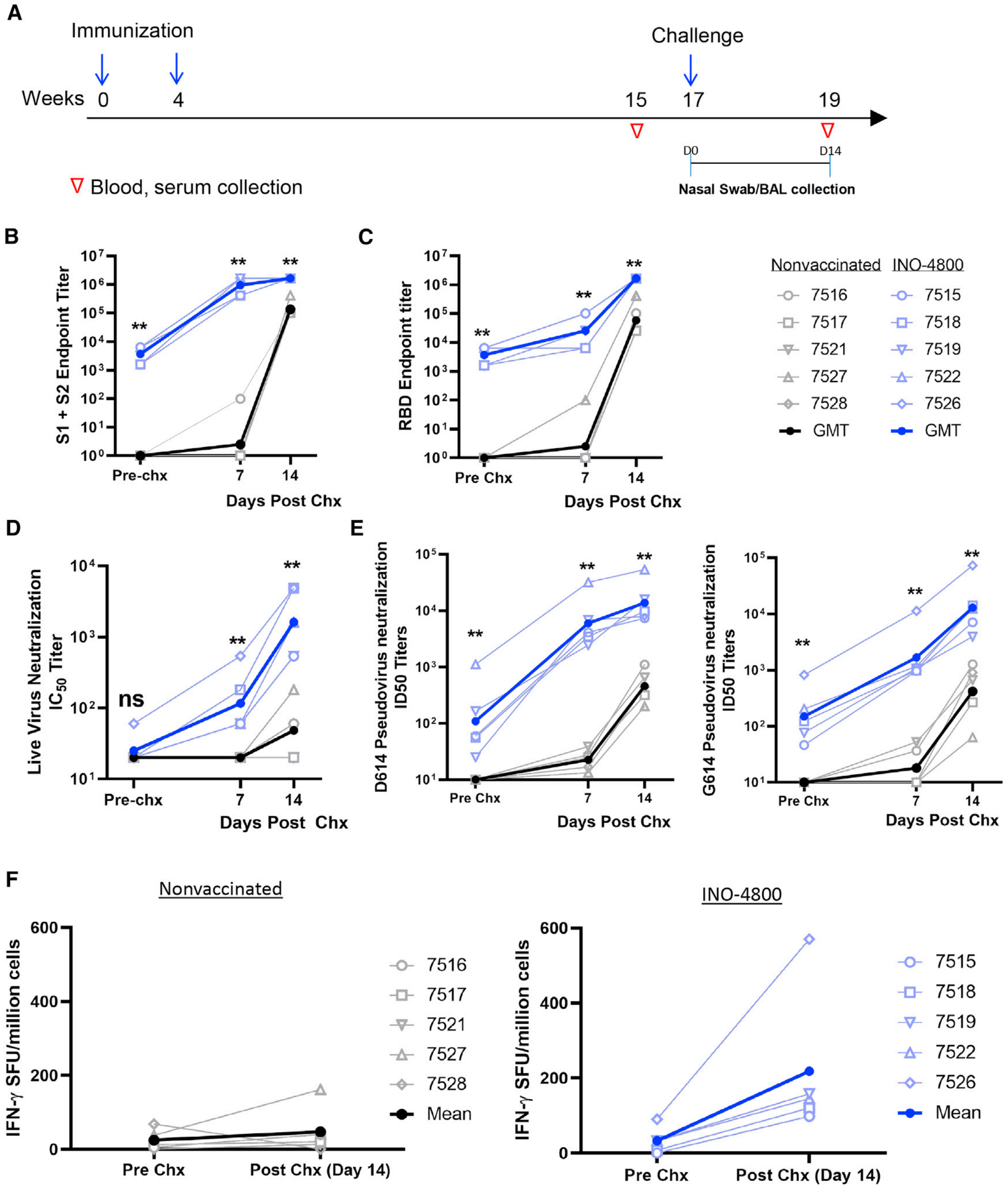

Figure 3. Recall of immune responses to SARS-CoV-2 after viral challenge

(A) Study outline.

(B and C) IgG binding ELISA. SARS-CoV-2 S1+S2 (B) and RBD (C) protein antigen binding of IgG in diluted NHP sera collected prior to challenge and post-challenge in INO-4800-vaccinated (right panels) and naive animals (left panels).

(D) Live virus neutralization.

(E) Pseudo-neutralization assay showing the presence of SARS-CoV-2-specific neutralizing antibodies against the D614 and G614 variants of SARS-CoV-2 before and after viral challenge.

(F) T cell responses were analyzed pre- and post-challenge with SARS-CoV-2 virus by IFN-γ ELISpot in PBMCs stimulated with overlapping peptide pools spanning the SARS-CoV-2 spike protein. Individual animal responses are depicted by open symbols and filled symbols represent median values. $^{**}p < 0.01$ Mann-Whitney test.

See also Figures S3 and S4.

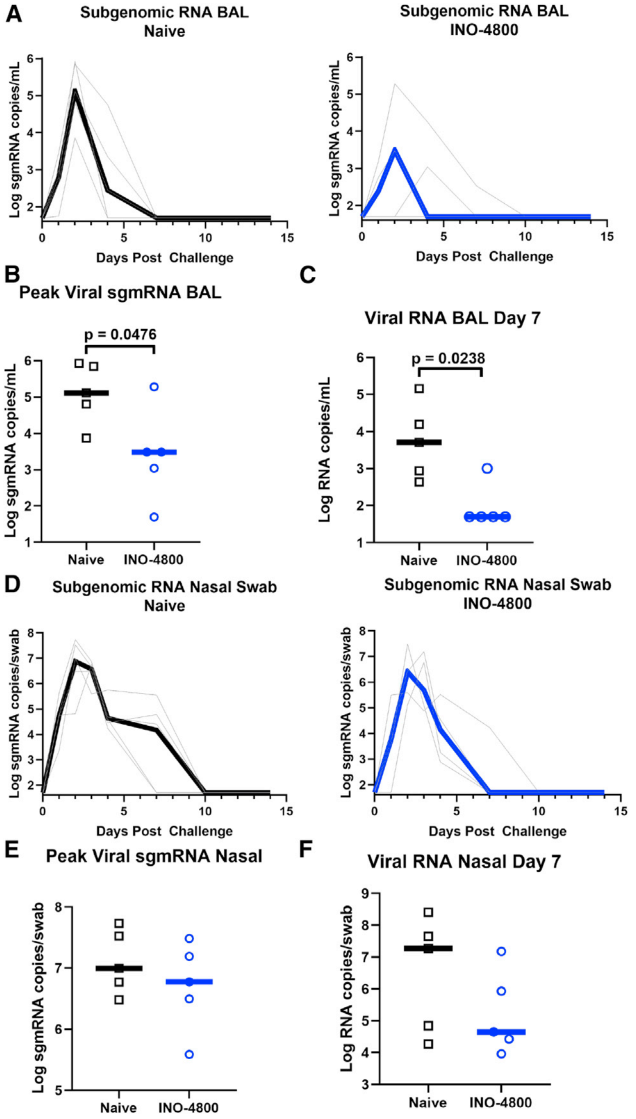

Figure 4. Viral loads in the BAL fluid and Nasal swabs after viral challenge

At week 17, naive and INO-4800-immunized (5 per group) rhesus macaques were challenged by intranasal and intratracheal administration of 1.1 × 10^4 PFU SARS-CoV-2 (US-WA1 isolate).

(A) Log sgmRNA copies/mL in BAL in naive (left panel) and INO-4800-vaccinated animals (right panel).

(B and C) Peak sgmRNA (B) and viral RNA (C) in BAL 7 days post-challenge.

(D) Log sgmRNA copies/mL in nasal swabs in naive (left panel) and INO-4800-vaccinated animals (right panel).

(E and F) Peak sgmRNA (E) and viral RNA (F) in nasal swabs 7 days post-challenge. Black and blue lines represent median values.

proteins through 14 days post-challenge. Faster antibody kinetics and significantly increased levels of neutralization titers against live SARS-CoV-2 and D614/G614 pseudoviruses were also observed in the INO-4800-vaccinated animals compared to nonvaccinated control animals, which only developed low neutralizing titers post-challenge (Figures 3D and 3E). Post-challenge serum antibody and pseudovirus neutralization titers from immunized NHPs were above the titers measured from human SARS-CoV-2 convalescent sera donors (Figures S3A and S3B).

Cellular responses were evaluated before and after challenge. At week 15, interferon (IFN)-γ ELISpot responses had returned to near baseline levels (~33 SFU/million cells average) (Figure 2A). Following challenge, T cell responses increased for all animals in the INO-4800-vaccinated group (~218 SFU/million cells average) compared to the nonvaccinated control animals, where the T cell responses were relatively low prior to challenge (~25 SFU/million cells average) and remained relatively unchanged (~48 SFU/million) post-challenge (Figure 3F).

Respiratory tract viral loads following SARS-CoV-2 challenge

At earlier time points post-challenge, viral mRNA detection does not discriminate between input challenge inoculum and active infection, while subgenomic (sgmRNA) levels are more likely representative of active cellular SARS-CoV-2 virion replication.[25,39,41] Therefore, SARS-CoV-2 sgmRNA was measured in unvaccinated control and INO-4800-vaccinated macaques following challenge (Figure 4A). Peak viral sgmRNA loads in the BAL were measured to be lower in the INO-4800-vaccinated group (median 3,060 sgmRNA copies/mL) as compared to nonvaccinated controls (median 130,000 sgmRNA copies/mL, p = 0.0476) (Figure 4B), concurrent with lower viral RNA loads at day 7 post-challenge (median 242 sgmRNA copies/mL in vaccinated NHPs and 33,418 sgmRNA copies/mL in control animals, p = 0.0238) (Figure 4C). While sgmRNA was detected in the nasal swabs of both the control and INO-4800-vaccinated animals (Figures 4D–4F), the median difference in viral mRNA levels trended downward in INO-4800-vaccinated animals at day 7 by more than 2 logs (Figure 4F), and clearance was observed by this time point for 4/5 vaccinated animals compared to 2/5 nonvaccinated animals. The reduced viral loads following exposure to SARS-CoV-2 infection at 17 weeks after immunization support a durable impact of INO-4800 on virus control.

DISCUSSION

The COVID-19 pandemic continues to devastate global health creating a destabilizing environment. Although several vaccines are available through EUA, the need for more preventative vaccine approaches for SARS-CoV-2 remains significant especially in LMICs. Acute SARS-CoV-2 challenge studies in NHPs receiving different vaccines have been reported.[18–23,25] These studies describe reduction in viral loads in the lower airways, with some studies indicating lower viral loads in the nose by IM delivered vaccines targeting full-length spike Ag. However, the study of protective efficacy at later time points post-immunization following contraction of the acute phase immune response has been limited. This is also true for prior vaccine studies in NHP MERS or SARS challenge models.[15,17,42] Accordingly, understanding the durability after vaccination of the immune recall response to coronavirus challenge is of importance.

In a recent clinical study, we reported that ID delivery of an HIV vaccine prototype was dose-sparing and demonstrated increased tolerability and improved immune potency compared

to IM DNA delivery.[43] We also demonstrated protective efficacy of a dose-sparing, 2-injection regimen following ID delivery of a MERS Spike DNA vaccine.[44] In the current study, we evaluated the durability of an ID-delivered full-length Spike antigen DNA vaccine (INO-4800) in rhesus macaques. The vaccine induced broad Spike binding antibodies to multiple regions of the antigen, as well as rapid induction of potent neutralizing antibodies, and cellular immune responses. INO-4800 vaccination also elicited cross-reactive humoral and cellular responses to the SARS-CoV S protein. We further demonstrated an impact on viral load after SARS-CoV-2 challenge more than 3 months post-final immunization, observing establishment of anamnestic immune responses and reduced viral loads in vaccinated macaques.

Other COVID-19 DNA vaccines have been evaluated in NHPs. Yu et al.[24] evaluated a full-length Spike antigen DNA vaccine candidate, similar to INO-4800. Administration of 2 × 5 mg IM doses to NHPs[25] followed by challenge at 3 weeks following the second immunization led to decreased BAL viral loads and a trend to lower nasal swab subgenomic viral loads. Here, we evaluated challenge outcome 13 weeks following delivery of a 2 × 1 mg dose by ID administration of INO-4800 in the same challenge model. In this study, we observed vaccine induction of durable immunity, with impact on viral replication following challenge 13 weeks post-final immunization. Reduced sgmRNA viral loads in the lower lung were observed. A trend toward lower viral mRNA loads was also observed in the nose at day 7 post-challenge in the INO-4800-vaccinated animals. These data and others[36] support that immunization and subsequent expansion of pre-existing antigen-specific T cells from such a vaccine in humans might protect from severe disease in the lung as well as limit the duration of viral shedding in the nasal cavity, thus possibly lowering viral transmission.

The initial viral loads detected in control unvaccinated animals in this study were approximately 2 logs higher (10^9 PFU/swab in 4/5 NHPs on day 1 post-challenge) than in similar published studies performed under identical conditions ($\sim10^7$ PFU/swab).[24] Viral replication in the respiratory tract of control unvaccinated animals was at the same level or higher than other studies using higher viral inoculum doses.[21,45,46] Challenge inoculums are frequently employed at higher doses than those experienced upon natural exposure to ensure take of infection; however, such a high-dose challenge may artificially reduce the impact of potentially protective vaccines and interventions.[47,48] For example, Wölfel et al.[41] reported nasal titers in patients of an average 6.76×10^5 copies/swab days 1–5 following onset of symptoms. These viral titers are significantly lower than in our NHP challenge ($>10^7$ copies/swab total virus). Despite these limitations, this study demonstrated significant reduction in peak BAL sgmRNA and overall viral mRNA months after final vaccination.

The current study was not designed to address the theoretical concern for vaccine enhanced disease. However, we did not observe an increase in viral replication in the vaccinated animals compared to controls, and no clinical signals such as respiratory distress, changes in heart rates or oxygen saturation levels, temperature spikes, or significant weight loss. Independent NHP studies that included lung imaging, histopathology, and immunopathology have been performed, and VED was not observed after challenge of animals receiving a single or two doses of INO-4800.[49]

T cell immunity has been shown to be an important mediator of protection against betacoronaviruses, and recent studies have specifically identified a role in protection against COVID-19 disease.[26,28,29,50] Sekine et al.[28] reported SARS-CoV-2-reactive T cells in asymptomatic and mild COVID-19 convalescent patients who were antibody seronegative. These results from convalescent patient studies collectively suggest that vaccine candidates that could generate balanced humoral and cellular immune responses could be important in providing protection against COVID-19 disease. Our study and other published reports show that DNA vaccination with candidates targeting the full-length SARS-CoV-2 spike protein likely increase the availability of T cell immunodominant epitopes leading to a broader and more potent immune response, compared to partial domains and truncated immunogens.[24] In this study, we also observe T cell cross-reactivity to SARS-CoV-1. Further evaluation of these shared responses is likely of interest and may inform development of vaccines targeting related betacoronaviruses. Long-lived SARS-CoV specific T cells have been reported.[34,35] Additional studies of SARS-CoV-2-infected patients will provide important insight toward immunity and long-term protection against COVID-19 disease.[51–53]

In addition to T cells, we demonstrated that INO-4800 induced durable antibody responses that rapidly increased following SARS-CoV-2 challenge. This increase was significantly higher than the generation of neutralizing antibodies in unvaccinated animals and likely contributed to viral control. Importantly, the vaccine induced robust neutralizing antibody responses against both D614 and G614 SARS-CoV-2 variants. While the D/G 614 site is outside the RBD, it has been suggested that this shift has the potential to impact vaccine-elicited antibodies[54] and possibly virus infectivity. Our data support induction of comparable neutralization titers between D614 and G614 variants and that these responses are similarly recalled following SARS-CoV-2 challenge. We additionally observe a positive relationship between vaccine-induced antibody binding to the RBD and neutralizing antibody titers (Figure S1C, R^2 = 0.69). Furthermore, sera from vaccine NHPs exhibited significant ADCP and ADCD activity, both associated with innate immune effector functions that have the potential to contribute toward vaccine efficacy.

Overall, the current challenge study provides a snapshot of anamnestic protective efficacy several months post-vaccination with a small cohort of animals. The data support further study of the SARS-CoV-2 DNA vaccine candidate INO-4800, which is currently in clinical trials. The persistence of SARS-CoV-2 immunity following natural infection is unknown, and recent studies suggest natural immunity may be short lived.[32] Given that many people exhibit asymptomatic or mild disease and recover without developing significant antibody responses, additional study of vaccines that induce immunological memory for T and B cell responses is clearly of interest. The immune responses and protection induced by simple ID delivery of INO-4800 are promising and provide important information to advancing SARS-CoV-2 vaccine development.

Limitations of the study

In the current study, there were several limitations that should be mentioned. The time between final immunization and live virus challenge, along with the presence of increased binding and neutralizing antibodies post-challenge, support the induction of immunological memory. However, identification of SARS-CoV-2-specific memory B cell responses were not evaluated as this was beyond the scope of the current study, and there was limited availability of SARS-CoV-2-specific reagents at the time this study was performed. It should also be noted that, at the time this study was conducted, the G614 strain, was the most prevalent variant of concern and the USA-WA1/2020 isolate was widely used by multiple groups for viral challenge.[21,22,38] We have since performed pseudoneutralization assays with recent VOC, including alpha, beta, gamma, and delta SARS-CoV-2 viruses to further demonstrate the breadth of protection against emerging strains in INO-4800-vaccinated subjects[55] and in NHPs (J.N.W., B. Schouest, A.P., E.L.R., K.S., E.P., I.M., E.N.G., M.P., V.A., A.D., D.E., Z.E., B. Nguyen, D. Frase, F.I.Z., A. Kulkarni, A.G., J.J.K., L.M.H., S.J.R., T.R.F.S., D.B.W., and K.B., unpublished data) following a similar treatment regimen as the current study. These data are included as part of a subsequent manuscript. A histopathological assessment of the lung tissue was planned following challenge to address the presence of virus-infected cells, but the samples were damaged during transport. In light of this, the viral load analysis indicates that all NHPs were infected but supports that vaccine-induced immunity likely contributed to lower viral loads in the immunized NHPs, potentially controlling disease progression. Similar observations are being reported with the current EUA COVID-19 vaccines with an increase of breakthrough infections being reported as the duration of time after vaccination extends but still strong control against disease progression and hospitalizations. However, in this study the lack of clinical symptoms in the NHP model did not allow for a correlation between disease and reduction of viral titers to be made in the collected BAL samples. Other INO-4800 NHP studies have now been reported with shorter durations of time lapse between vaccination and challenge and provide a histopathological assessment of the virus-infected tissue.[49] Future studies are being planned to address timing of challenge following the last vaccination and to provide a histopathological assessment of the virus-infected tissue.

STAR★METHODS

Detailed methods are provided in the online version of this paper and include the following:

- KEY RESOURCES TABLE
- RESOURCE AVAILABILITY
  - Lead contact
  - Materials availability
  - Data and code availability
- EXPERIMENTAL MODEL AND SUBJECT DETAILS
  - Cell lines
  - In vivo animal studies animals
- METHOD DETAILS
  - DNA vaccine, INO-4800
  - Peripheral blood mononuclear cell isolation
  - IFN-γ enzyme-linked immunospot
  - Antigen binding ELISA
  - ACE2 competition ELISA non-human primates
  - Flow cytometry-based ACE2 receptor binding inhibition assay
  - Pseudovirus neutralization assay
  - Plaque reduction neutralization test
  - Antibody-dependent cellular phagocytosis
  - Antibody-dependent complement deposition
  - Viral RNA assay
  - Subgenomic mRNA assay
- QUANTIFICATION AND STATISTICAL ANALYSIS

SUPPLEMENTAL INFORMATION

Supplemental information can be found online at https://doi.org/10.1016/j.xcrm.2021.100420.

ACKNOWLEDGMENTS

The studies described in this manuscript were funded by a grant from the Coalition for Epidemic Preparedness Innovations (CEPI). The authors would like to additionally thank Olivia Bedoya, Gloria Mendez, Francisco Vega Vega, and Jon Schantz at Inovio Pharmaceuticals for their assistance, and Jack Greenhouse and Tammy Putmon-Taylor at Bioqual for their expert assistance.

AUTHOR CONTRIBUTIONS

Conceptualization, A.P., J.N.W., J.J.K., S.J.R., T.R.F.S., D.B.W., and K.E.B.; methodology, A.P., J.N.W., E.L.R., K.S., E.P., E.N.G., and D.W.K.; investigation, A.P., J.N.W., E.L.R., K.S., E.P., E.N.G., I.M., M.P., Z.E., S.N.W., O.S.A., D.G., P.B., A.D., Z.X., D.E., S.M.R., L.P., K.Y.K., A.C., N.C., B.F., E.T-R., A.D., J.C., A.G., N.J.T., V.M.A., Y.D., F.I.Z., M.A.-M., and D.W.K.; resources, K.M., D.W.K., H.A., and M.G.L.; writing – original draft, A.P., J.N.W., and T.R.F.S.; writing – review & editing, A.P., J.N.W., E.L.R., E.P., E.N.G., M.P., P.B., J.J.K., L.M.H., S.J.R., T.R.F.S., and D.B.W.; supervision: A.P., J.N.W., S.R., T.R.F.S., D.B.W., and K.E.B.; project administration: A.P., J.N.W., J.J.K., L.M.H., S.J.R., T.R.F.S., D.B.W., and K.E.B.; funding acquisition: J.J.K., L.M.H., D.B.W., and K.E.B.

DECLARATION OF INTERESTS

A.P., E.L.R., E.P., E.N.G., M.P., S.N.W., P.B., Z.X., S.M.R., K.Y.K., N.C., E.T-R., J.C., N.J.T., K.M., and D.K.W. declare no competing interests. J.N.W., K.S., I.M., Z.E., D.G., A.D., D.E., A.G., V.M.A., J.J.K., L.M.H., S.J.R., T.R.F.S., and K.E.B. are employees of Inovio Pharmaceuticals and as such receive salary and benefits, including ownership of stock and stock options, from the company. D.B.W. discloses the following paid associations with commercial partners: GeneOne (Consultant), Geneos (Advisory Board), Astrazeneca (Advisory Board, Speaker), Inovio (BOD, SRA, Stock), Pfizer (Speaker), Merck (Speaker), Sanofi (Advisory Board), BBI (Advisory Board).

Received: November 25, 2020
Revised: May 25, 2021
Accepted: September 22, 2021
Published: September 28, 2021

REFERENCES

1. Lu, R., Zhao, X., Li, J., Niu, P., Yang, B., Wu, H., Wang, W., Song, H., Huang, B., Zhu, N., et al. (2020). Genomic characterisation and epidemiology of 2019 novel coronavirus: implications for virus origins and receptor binding. Lancet *395*, 565–574.

2. Huang, C., Wang, Y., Li, X., Ren, L., Zhao, J., Hu, Y., Zhang, L., Fan, G., Xu, J., Gu, X., et al. (2020). Clinical features of patients infected with 2019 novel coronavirus in Wuhan, China. Lancet *395*, 497–506.
3. Xu, Z., Shi, L., Wang, Y., Zhang, J., Huang, L., Zhang, C., Liu, S., Zhao, P., Liu, H., Zhu, L., et al. (2020). Pathological findings of COVID-19 associated with acute respiratory distress syndrome. Lancet Respir. Med. *8*, 420–422.
4. Thanh Le, T., Andreadakis, Z., Kumar, A., Gómez Román, R., Tollefsen, S., Saville, M., and Mayhew, S. (2020). The COVID-19 vaccine development landscape. Nat. Rev. Drug Discov. *19*, 305–306.
5. FDA (2020). COVID-19 Vaccines (US Department of Health and Human Services, Food and Drug Administration).
6. Dooling, K., McClung, N., Chamberland, M., Marin, M., Wallace, M., Bell, B.P., Lee, G.M., Talbot, H.K., Romero, J.R., and Oliver, S.E. (2020). The Advisory Committee on Immunization Practices' Interim Recommendation for Allocating Initial Supplies of COVID-19 Vaccine - United States, 2020. MMWR Morb. Mortal. Wkly. Rep. *69*, 1857–1859.
7. Oliver, S.E., Gargano, J.W., Marin, M., Wallace, M., Curran, K.G., Chamberland, M., McClung, N., Campos-Outcalt, D., Morgan, R.L., Mbaeyi, S., et al. (2021). The Advisory Committee on Immunization Practices' Interim Recommendation for Use of Moderna COVID-19 Vaccine - United States, December 2020. MMWR Morb. Mortal. Wkly. Rep. *69*, 1653–1656.
8. Robbiani, D.F., Gaebler, C., Muecksch, F., Lorenzi, J.C.C., Wang, Z., Cho, A., Agudelo, M., Barnes, C.O., Gazumyan, A., Finkin, S., et al. (2020). Convergent antibody responses to SARS-CoV-2 in convalescent individuals. Nature *584*, 437–442.
9. Hoffmann, M., Kleine-Weber, H., Schroeder, S., Krüger, N., Herrler, T., Erichsen, S., Schiergens, T.S., Herrler, G., Wu, N.H., Nitsche, A., et al. (2020). SARS-CoV-2 Cell Entry Depends on ACE2 and TMPRSS2 and Is Blocked by a Clinically Proven Protease Inhibitor. Cell *181*, 271–280.
10. Cantuti-Castelvetri, L., Ojha, R., Pedro, L.D., Djannatian, M., Franz, J., Kuivanen, S., van der Meer, F., Kallio, K., Kaya, T., Anastasina, M., et al. (2020). Neuropilin-1 facilitates SARS-CoV-2 cell entry and infectivity. Science *370*, 856–860.
11. Daly, J.L., Simonetti, B., Klein, K., Chen, K.E., Williamson, M.K., Antón-Plágaro, C., Shoemark, D.K., Simón-Gracia, L., Bauer, M., Hollandi, R., et al. (2020). Neuropilin-1 is a host factor for SARS-CoV-2 infection. Science *370*, 861–865.
12. Soresina, A., Moratto, D., Chiarini, M., Paolillo, C., Baresi, G., Focà, E., Bezzi, M., Baronio, B., Giacomelli, M., and Badolato, R. (2020). Two X-linked agammaglobulinemia patients develop pneumonia as COVID-19 manifestation but recover. Pediatr. Allergy Immunol. *31*, 565–569.
13. Du, L., He, Y., Zhou, Y., Liu, S., Zheng, B.J., and Jiang, S. (2009). The spike protein of SARS-CoV–a target for vaccine and therapeutic development. Nat. Rev. Microbiol. *7*, 226–236.
14. Roper, R.L., and Rehm, K.E. (2009). SARS vaccines: where are we? Expert Rev. Vaccines *8*, 887–898.
15. Muthumani, K., Falzarano, D., Reuschel, E.L., Tingey, C., Flingai, S., Villarreal, D.O., Wise, M., Patel, A., Izmirly, A., Aljuaid, A., et al. (2015). A synthetic consensus anti-spike protein DNA vaccine induces protective immunity against Middle East respiratory syndrome coronavirus in nonhuman primates. Sci. Transl. Med. *7*, 301ra132.
16. Liu, R., Wang, J., Shao, Y., Wang, X., Zhang, H., Shuai, L., Ge, J., Wen, Z., and Bu, Z. (2018). A recombinant VSV-vectored MERS-CoV vaccine induces neutralizing antibody and T cell responses in rhesus monkeys after single dose immunization. Antiviral Res. *150*, 30–38.
17. van Doremalen, N., Haddock, E., Feldmann, F., Meade-White, K., Bushmaker, T., Fischer, R.J., Okumura, A., Hanley, P.W., Saturday, G., Edwards, N.J., et al. (2020). A single dose of ChAdOx1 MERS provides protective immunity in rhesus macaques. Sci. Adv. *6*, eaba8399.
18. Gao, Q., Bao, L., Mao, H., Wang, L., Xu, K., Yang, M., Li, Y., Zhu, L., Wang, N., Lv, Z., et al. (2020). Development of an inactivated vaccine candidate for SARS-CoV-2. Science *369*, 77–81.
19. van Doremalen, N., Lambe, T., Spencer, A., Belij-Rammerstorfer, S., Purushotham, J.N., Port, J.R., Avanzato, V.A., Bushmaker, T., Flaxman, A., Ulaszewska, M., et al. (2020). ChAdOx1 nCoV-19 vaccine prevents SARS-CoV-2 pneumonia in rhesus macaques. Nature *586*, 578–582.
20. Mercado, N.B., Zahn, R., Wegmann, F., Loos, C., Chandrashekar, A., Yu, J., Liu, J., Peter, L., McMahan, K., Tostanoski, L.H., et al. (2020). Single-shot Ad26 vaccine protects against SARS-CoV-2 in rhesus macaques. Nature *586*, 583–588.
21. Corbett, K.S., Flynn, B., Foulds, K.E., Francica, J.R., Boyoglu-Barnum, S., Werner, A.P., Flach, B., O'Connell, S., Bock, K.W., Minai, M., et al. (2020). Evaluation of the mRNA-1273 Vaccine against SARS-CoV-2 in Nonhuman Primates. N. Engl. J. Med. *383*, 1544–1555.
22. Vogel, A.B., Kanevsky, I., Che, Y., Swanson, K.A., Muik, A., Vormehr, M., Kranz, L.M., Walzer, K.C., Hein, S., Güler, A., et al. (2021). BNT162b vaccines protect rhesus macaques from SARS-CoV-2. Nature *592*, 283–289.
23. Guebre-Xabier, M., Patel, N., Tian, J.H., Zhou, B., Maciejewski, S., Lam, K., Portnoff, A.D., Massare, M.J., Frieman, M.B., Piedra, P.A., et al. (2020). NVX-CoV2373 vaccine protects cynomolgus macaque upper and lower airways against SARS-CoV-2 challenge. Vaccine *38*, 7892–7896.
24. Yu, J., Tostanoski, L.H., Peter, L., Mercado, N.B., McMahan, K., Mahrokhian, S.H., Nkolola, J.P., Liu, J., Li, Z., Chandrashekar, A., et al. (2020). DNA vaccine protection against SARS-CoV-2 in rhesus macaques. Science *369*, 806–811.
25. Chi, X., Yan, R., Zhang, J., Zhang, G., Zhang, Y., Hao, M., Zhang, Z., Fan, P., Dong, Y., Yang, Y., et al. (2020). A neutralizing human antibody binds to the N-terminal domain of the Spike protein of SARS-CoV-2. Science *369*, 650–655.
26. Sun, J., Zhuang, Z., Zheng, J., Li, K., Wong, R.L., Liu, D., Huang, J., He, J., Zhu, A., Zhao, J., et al. (2020). Generation of a Broadly Useful Model for COVID-19 Pathogenesis, Vaccination, and Treatment. Cell *182*, 734–743.
27. Peng, Y., Mentzer, A.J., Liu, G., Yao, X., Yin, Z., Dong, D., Dejnirattisai, W., Rostron, T., Supasa, P., Liu, C., et al.; Oxford Immunology Network Covid-19 Response T cell Consortium; ISARIC4C Investigators (2020). Broad and strong memory $CD4^+$ and $CD8^+$ T cells induced by SARS-CoV-2 in UK convalescent individuals following COVID-19. Nat. Immunol. *21*, 1336–1345.
28. Sekine, T., Perez-Potti, A., Rivera-Ballesteros, O., Strålin, K., Gorin, J.-B., Olsson, A., Llewellyn-Lacey, S., Kamal, H., Bogdanovic, G., Muschiol, S., et al. (2020). Robust T cell immunity in convalescent individuals with asymptomatic or mild COVID-19. Cell *183*, 158–168.
29. Channappanavar, R., Zhao, J., and Perlman, S. (2014). T cell-mediated immune response to respiratory coronaviruses. Immunol. Res. *59*, 118–128.
30. Gretebeck, L.M., and Subbarao, K. (2015). Animal models for SARS and MERS coronaviruses. Curr. Opin. Virol. *13*, 123–129.
31. Payne, D.C., Smith-Jeffcoat, S.E., Nowak, G., Chukwuma, U., Geibe, J.R., Hawkins, R.J., Johnson, J.A., Thornburg, N.J., Schiffer, J., Weiner, Z., et al.; CDC COVID-19 Surge Laboratory Group (2020). SARS-CoV-2 Infections and Serologic Responses from a Sample of U.S. Navy Service Members - USS Theodore Roosevelt, April 2020. MMWR Morb. Mortal. Wkly. Rep. *69*, 714–721.
32. Long, Q.X., Tang, X.J., Shi, Q.L., Li, Q., Deng, H.J., Yuan, J., Hu, J.L., Xu, W., Zhang, Y., Lv, F.J., et al. (2020). Clinical and immunological assessment of asymptomatic SARS-CoV-2 infections. Nat. Med. *26*, 1200–1204.
33. Tang, F., Quan, Y., Xin, Z.T., Wrammert, J., Ma, M.J., Lv, H., Wang, T.B., Yang, H., Richardus, J.H., Liu, W., and Cao, W.C. (2011). Lack of peripheral memory B cell responses in recovered patients with severe acute respiratory syndrome: a six-year follow-up study. J. Immunol. *186*, 7264–7268.
34. Yang, L.T., Peng, H., Zhu, Z.L., Li, G., Huang, Z.T., Zhao, Z.X., Koup, R.A., Bailer, R.T., and Wu, C.Y. (2006). Long-lived effector/central memory T-

cell responses to severe acute respiratory syndrome coronavirus (SARS-CoV) S antigen in recovered SARS patients. Clin. Immunol. *120*, 171–178.

35. Le Bert, N., Tan, A.T., Kunasegaran, K., Tham, C.Y.L., Hafezi, M., Chia, A., Chng, M.H.Y., Lin, M., Tan, N., Linster, M., et al. (2020). SARS-CoV-2-specific T cell immunity in cases of COVID-19 and SARS, and uninfected controls. Nature *584*, 457–462.
36. McMahan, K., Yu, J., Mercado, N.B., Loos, C., Tostanoski, L.H., Chandrashekar, A., Liu, J., Peter, L., Atyeo, C., Zhu, A., et al. (2021). Correlates of protection against SARS-CoV-2 in rhesus macaques. Nature *590*, 630–634.
37. Smith, T.R.F., Patel, A., Ramos, S., Elwood, D., Zhu, X., Yan, J., Gary, E.N., Walker, S.N., Schultheis, K., Purwar, M., et al. (2020). Immunogenicity of a DNA vaccine candidate for COVID-19. Nat. Commun. *11*, 2601.
38. Korber, B., Fischer, W.M., Gnanakaran, S., Yoon, H., Theiler, J., Abfalterer, W., Hengartner, N., Giorgi, E.E., Bhattacharya, T., Foley, B., et al.; Sheffield COVID-19 Genomics Group (2020). Tracking Changes in SARS-CoV-2 Spike: Evidence that D614G Increases Infectivity of the COVID-19 Virus. Cell *182*, 812–827.e19.
39. Chandrashekar, A., Liu, J., Martinot, A.J., McMahan, K., Mercado, N.B., Peter, L., Tostanoski, L.H., Yu, J., Maliga, Z., Nekorchuk, M., et al. (2020). SARS-CoV-2 infection protects against rechallenge in rhesus macaques. Science *369*, 812–817.
40. Walker, S.N., Chokkalingam, N., Reuschel, E.L., Purwar, M., Xu, Z., Gary, E.N., Kim, K.Y., Helble, M., Schultheis, K., Walters, J., et al. (2020). SARS-CoV-2 assays to detect functional antibody responses that block ACE2 recognition in vaccinated animals and infected patients. J. Clin. Microbiol. *58*, e01533-20.
41. Wölfel, R., Corman, V.M., Guggemos, W., Seilmaier, M., Zange, S., Müller, M.A., Niemeyer, D., Jones, T.C., Vollmar, P., Rothe, C., et al. (2020). Virological assessment of hospitalized patients with COVID-2019. Nature *581*, 465–469.
42. Roberts, A., Lamirande, E.W., Vogel, L., Jackson, J.P., Paddock, C.D., Guarner, J., Zaki, S.R., Sheahan, T., Baric, R., and Subbarao, K. (2008). Animal models and vaccines for SARS-CoV infection. Virus Res. *133*, 20–32.
43. De Rosa, S.C., Edupuganti, S., Huang, Y., Han, X., Elizaga, M., Swann, E., Polakowski, L., Kalams, S.A., Keefer, M.C., Maenza, J., et al.; HIV Vaccine Trials Network (HVTN) 098 Study Team (2020). Robust antibody and cellular responses induced by DNA-only vaccination for HIV. JCI Insight *5*, 137079.
44. Patel, A., Reuschel, E.L., Xu, Z., Zaidi, F.I., Kim, K.Y., Scott, D.P., Mendoza, J., Ramos, S., Stoltz, R., Feldmann, F., et al. (2021). Intradermal delivery of a synthetic DNA vaccine protects macaques from Middle East respiratory syndrome coronavirus. JCI Insight *6*, 146082.
45. Tian, J.-H., Patel, N., Haupt, R., Zhou, H., Weston, S., Hammond, H., Lague, J., Portnoff, A.D., Norton, J., Guebre-Xabier, M., et al. (2020). SARS-CoV-2 spike glycoprotein vaccine candidate NVX-CoV2373 elicits immunogenicity in baboons and protection in mice. bioRxiv. https://doi.org/10.1101/2020.06.29.178509.
46. Vogel, A.B., Kanevsky, I., Che, Y., Swanson, K.A., Muik, A., Vormehr, M., Kranz, L.M., Walzer, K.C., Hein, S., Güler, A., et al. (2020). A prefusion SARS-CoV-2 spike RNA vaccine is highly immunogenic and prevents lung infection in non-human primates. bioRxiv. https://doi.org/10.1101/2020.09.08.280818.
47. Innis, B.L., Scorza, F.B., Blum, J.S., Jain, V.K., Aguilar, A.O., Post, D.J., Roberts, P.C., Wairagkar, N., White, J., and Bresee, J. (2019). Convening on the influenza human viral challenge model for universal influenza vaccines, Part 2: Methodologic considerations. Vaccine *37*, 4830–4834.
48. Durudas, A., Chen, H.L., Gasper, M.A., Sundaravaradan, V., Milush, J.M., Silvestri, G., Johnson, W., Giavedoni, L.D., and Sodora, D.L. (2011). Differential innate immune responses to low or high dose oral SIV challenge in Rhesus macaques. Curr. HIV Res. *9*, 276–288.
49. Gooch, K.E., Smith, T.R.F., Salguero, F.J., Fotheringham, S.A., Watson, R.J., Dennis, M.J., Handley, A., Humphries, H.E., Longet, S., Tipton, T., et al. (2021). One or two dose regimen of the SARS-CoV-2 synthetic DNA vaccine INO-4800 protects against respiratory tract disease burden in nonhuman primate challenge model. Vaccine *39*, 4885–4894.
50. Channappanavar, R., Fett, C., Zhao, J., Meyerholz, D.K., and Perlman, S. (2014). Virus-specific memory CD8 T cells provide substantial protection from lethal severe acute respiratory syndrome coronavirus infection. J. Virol. *88*, 11034–11044.
51. Wajnberg, A., Amanat, F., Firpo, A., Altman, D.R., Bailey, M.J., Mansour, M., McMahon, M., Meade, P., Mendu, D.R., Muellers, K., et al. (2020). Robust neutralizing antibodies to SARS-CoV-2 infection persist for months. Science *370*, 1227–1230.
52. Ma, T., Ryu, H., McGregor, M., Babcock, B., Neidleman, J., Xie, G., George, A.F., Frouard, J., Murray, V., Gill, G., et al. (2021). Protracted yet coordinated differentiation of long-lived SARS-CoV-2-specific CD8+ T cells during COVID-19 convalescence. J. Immunol. Published online August 13, 2021. https://doi.org/10.4049/jimmunol.2100465.
53. Dan, J.M., Mateus, J., Kato, Y., Hastie, K.M., Yu, E.D., Faliti, C.E., Grifoni, A., Ramirez, S.I., Haupt, S., Frazier, A., et al. (2021). Immunological memory to SARS-CoV-2 assessed for up to 8 months after infection. Science *371*, eabf4063.
54. Korber, B., Fischer, W.M., Gnanakaran, S., Yoon, H., Theiler, J., Abfalterer, W., Hengartner, N., Giorgi, E.E., Bhattacharya, T., Foley, B., et al. (2020). Tracking changes in SARS-CoV-2 Spike: evidence that D614G increases infectivity of the COVID-19 virus. Cell *182*, 812–827.
55. Andrade, V.M., Christensen-Quick, A., Agnes, J., Tur, J., Reed, C., Kalia, R., Marrero, I., Elwood, D., Schultheis, K., Purwar, M., et al. (2021). INO-4800 DNA Vaccine Induces Neutralizing Antibodies and T cell Activity Against Global SARS-CoV-2 Variants. bioRxiv, 2021.2004.2014.439719.
56. Abbink, P., Mercado, N.B., Nkolola, J.P., Peterson, R.L., Tuyishime, H., McMahan, K., Moseley, E.T., Borducchi, E.N., Chandrashekar, A., Bondzie, E.A., et al. (2019). Lack of therapeutic efficacy of an antibody to $\alpha_4\beta_7$ in SIVmac251-infected rhesus macaques. Science *365*, 1029–1033.

STAR★METHODS

KEY RESOURCES TABLE

REAGENT OR RESOURCE	SOURCE	IDENTIFIER
**Antibodies**		
anti-monkey IgG conjugated to horseradish peroxidase	Southern Biotech	Cat# 4700-05; RRID: AB_2796069
PolyRab anti-His antibody	ThermoFisher	Cat# PA1-983B; RRID: AB_1069891
goat anti-mouse IgG H^{+}L HRP	Bethyl Laboratories	Cat# A90-116P; RRID: AB_67183
surelight® APC conjugated anti-his antibody	Abcam	Cat# Ab72579; RRID: AB_1267597
**Bacterial and virus strains**		
SARS-CoV-2 USA-WA1/2020	BEI Resources	NR-52281
**Biological samples**		
Peripheral blood mononuclear cells	This paper	N/A
Bronchoalveolar lavage	This paper	N/A
**Chemicals, peptides, and recombinant proteins**		
SARS-CoV-2 S1+S2 ECD protein	Sino Biological	40589-V08B1
SARS-CoV-2 S1 protein	Sino Biological	40591-V08H
SARS-CoV-2 S2 protein	Sino Biological	40590-V08B
SARS-CoV-2 RBD protein	Sino Biological	40595-V05H
1-step TMB ultra substrate	ThermoFisher	37574
GeneJammer	Agilent	204130
Britelite plus luminescence reporter gene assay system	PerkinElmer	6066769
**Critical commercial assays**		
Monkey IFN-γ ELISpotPro	Mabtech	3421M-2APW-10
Cador pathogen HT kit	QIAGEN	950067
**Experimental models: Cell lines**		
ACE2-CHO cell line	Creative Biolabs	VCeL-Wyb019
ACE2-293T	This paper	n/A
**Experimental models: Organisms/strains**		
Chinese rhesus macaques	Bioqual	N/A
**Oligonucleotides**		
2019-nCoV_N1-F:5′-GACCCCAAA ATCAGCGAAAT-3′	Bioqual	Ref#41
2019-nCoV_N1-R: 5′-TCTGGTTACT GCCAGTTGAATCTG-3′	Bioqual	Ref#41
2019-nCoV_N1-P: 5′-FAM-ACCCCGCAT TACGTTTGGTGGACC-BHQ1-3′	Bioqual	Ref#41
**Recombinant DNA**		
DNA vaccine, INO-4800	Smith et al., 2020 Nat Commun	N/A
pNL4-3.Luc.R-E- plasmid	NIH AIDS reagent	N/A
**Software and algorithms**		
GraphPad Prism 8.0	GraphPad Software	N/A

RESOURCE AVAILABILITY

Lead contact

Further information and requests for resources and reagents should be directed to and will be fulfilled by the Lead Contact, Kate E. Broderick (kate.broderick@inovio.com).

Materials availability

Requests for resources and reagents should be directed to and will be fulfilled by the Lead Contact, Kate E. Broderick. This includes plasmids, cell lines, and pseudoviruses. All reagents will be made available on request following completion of a Material Transfer Agreement.

Data and code availability

- All data for this study are available without restriction from the Lead Contact upon request.
- This study did not generate code.
- Any additional information required to reanalyze the data reported in this study is available from the lead contact upon request.

EXPERIMENTAL MODEL AND SUBJECT DETAILS

Cell lines

ACE2-CHO and ACE2-293T cells were maintained in Dulbecco's Modified Eagles Medium (DMEM, GIBCO) at 37°C, 5% CO_2. All cell lines were tested to be mycoplasma negative.

In vivo animal studies animals

All rhesus macaque experiments were approved by the Institutional Animal Care and Use Committee at Bioqual (Rockville, Maryland), an Association for Assessment and Accreditation of Laboratory Animal Care (AAALAC) International accredited facility, IACUC# 18-072 (Study #SP2000012). Ten Chinese rhesus macaques (ranging from 4.55kg-5.55kg) were randomly assigned to be immunized (3 males and 2 females) or naive (2 males and 3 females). The sex and age in years old on day 0 for each animal were: 7515 (M, 3.9), 7516 (M, 3.6), 7517 (M, 3.8), 7518 (M, 3.7), 7519 (M, 3.8), 7521 (F, 3.9), 7522 (F, 3.6), 7526 (F, 3.5), 7527 (F, 3.8), 7528 (F, 3.7). Immunized macaques received two 1 mg injections of SARS-CoV-2 DNA vaccine, INO-4800, at week 0 and 4 by the minimally invasive ID-EP administration using the CELLECTRA 2000® Adaptive Constant Current Electroporation Device with a 3P array (Inovio Pharmaceuticals). At week 17, all animals were challenged with 1.2×10^8 VP (1.1×10^4 PFU) SARS-CoV-2 isolate USA-WA1/2020 (BEI Resources NR-52281). The following reagent was deposited by the Centers for Disease Control and Prevention and obtained through BEI Resources, NIAID, NIH: SARS-Related Coronavirus 2, Isolate USA-WA1/2020, NR-52281. Virus was administered as 1 mL by the intranasal route (0.5 mL in each nostril) and 1 mL by the intratracheal route. Nasal swabs were collected during the challenge period using Copan flocked swabs and placed into 1 mL PBS. Blood was collected at indicated pre-challenge and post-challenge time points to analyze blood chemistry and to isolate peripheral blood mononuclear cells (PBMC), and serum. Bronchoalveolar lavage was collected at Week 8 and on Days 1, 2, 4, 7 post challenge to assay lung viral loads. BAL from naive animals was run as control.

METHOD DETAILS

DNA vaccine, INO-4800

The highly optimized DNA sequence encoding SARS-CoV-2 IgE-spike was created using Inovio's proprietary *in silico* Gene Optimization Algorithm to enhance expression and immunogenicity.[37] The optimized DNA sequence was synthesized, digested with BamHI and XhoI, and cloned into the expression vector pGX0001 under the control of the human cytomegalovirus immediate-early promoter and a bovine growth hormone polyadenylation signal.

Peripheral blood mononuclear cell isolation

Blood was collected from each macaque into sodium citrate cell preparation tubes (CPT, BD Biosciences). The tubes were centrifuged to separate plasma and lymphocytes, according to the manufacturer's protocol. Samples were transported by same-day shipment on cold-packs from Bioqual to The Wistar Institute for PBMC isolation. PBMCs were washed and residual red blood cells were removed using ammonium-chloride-potassium (ACK) lysis buffer. Cells were counted using a ViCell counter (Beckman Coulter) and resuspended in RPMI 1640 (Corning), supplemented with 10% fetal bovine serum (Atlas), and 1% penicillin/streptomycin (GIBCO). Fresh cells were then plated for IFNγ ELISpot Assays and flow cytometry.

IFN-γ enzyme-linked immunospot

Monkey IFN-γ ELISpot assay was performed to detect cellular responses. Monkey IFN-γ ELISpotPro (alkaline phosphatase) plates (Mabtech, Sweden, Cat#3421M-2APW-10) were blocked for a minimum of 2 h with RPMI 1640 (Corning), supplemented with 10% FBS and 1% pen/strep (R10). Following PBMC isolation, 200 000 cells were added to each well in the presence of 1) peptide pools (15-mers with 9-mer overlaps) corresponding to the SARS-CoV-1, SARS-CoV-2, or MERS-CoV spike proteins (5ug/mL/well final concentration), 2) R10 with DMSO (negative control), or 3) anti-CD3 positive control (Mabtech, 1:1000 dilution). All samples were plated in triplicate. Plates were incubated overnight at 37°C, 5% CO_2. After 18-20 h, the plates were washed in PBS and spots

were developed according to the manufacturer's protocol. Spots were imaged using a CTL Immunospot plate reader and antigen-specific responses were determined by subtracting the number of spots in the R10+DMSO negative control well from the wells stimulated with peptide pools.

Antigen binding ELISA

Serum and BAL was collected at each time point was evaluated for binding titers. Ninety-six well immunosorbent plates (NUNC) were coated with 1ug/mL recombinant SARS-CoV-2 S1+S2 ECD protein (Sino Biological 40589-V08B1), S1 protein (Sino Biological 40591-V08H), S2 protein (Sino Biological 40590-V08B), or receptor-binding domain (RBD) protein (Sino Biological 40595-V05H) in PBS overnight at 4°C. ELISA plates were also coated with 1ug/mL recombinant SARS-CoV S1 protein (Sino Biological 40150-V08B1) and RBD protein (Sino Biological 40592-V08B) or MERS-CoV spike (Sino Biological 40069-V08B). Plates were washed four times with PBS + 0.05% Tween20 (PBS-T) and blocked with 5% skim milk in PBS-T (5% SM) for 90 min at 37°C. Sera or BAL from INO-4800 vaccinated and control macaques were serially diluted in 5% SM, added to the washed ELISA plates, and then incubated for 1 h at 37°C. Following incubation, plates were washed 4 times with PBS-T and an anti-monkey IgG conjugated to horseradish peroxidase (Southern Biotech 4700-05) 1 h at 37°C. Plates were washed 4 times with PBS-T and one-step TMB solution (Sigma) was added to the plates. The reaction was stopped with an equal volume of 2N sulfuric acid. Plates were read at 450nm and 570nm within 30 min of development using a BioTEK Synergy2 plate reader.

ACE2 competition ELISA non-human primates

96-well half area plates (Corning) were coated at room temperature for 3 h with 1 μg/mL PolyRab anti-His antibody (ThermoFisher, PA1-983B), followed by overnight blocking with blocking buffer containing 1x PBS, 5% SM, 1% FBS, and 0.2% Tween-20. The plates were then incubated with 10 μg/mL of His6x-tagged SARS-CoV-2, S1+S2 ECD (Sino Biological, 40589-V08B1) at room temperature for 1-2 h. NHP sera (Day 0 or Week 6) was serially diluted 3-fold with 1XPBS containing 1% FBS and 0.2% Tween and pre-mixed with huACE2-IgMu at constant concentration of 0.4ug/ml. The pre-mixture was then added to the plate and incubated at room temperature for 1-2 h. The plates were further incubated at room temperature for 1 h with goat anti-mouse IgG $H^{+}L$ HRP (A90-116P, Bethyl Laboratories) at 1:20,000 dilution followed by addition of one-step TMB ultra substrate (ThermoFisher) and then quenched with 1M H_2SO_4. Absorbance at 450nm and 570nm were recorded with BioTEK plate reader.

Flow cytometry-based ACE2 receptor binding inhibition assay

HEK293T cells stably expressing ACE2-GFP were generated using retroviral transduction. Following transduction, the cells were flow sorted based on GFP expression to isolate GFP positive cells. Single cell cloning was done on these cells to generate cell lines with equivalent expression of ACE2-GFP. To detect inhibition of spike binding to ACE2, 2.5 μg/ml S1+S2 ECD-his tagged (Sino Biological, Catalog #40589-V08B1) was incubated with serum collected from vaccinated animals at indicated time points and dilutions on ice for 60 min. This mixture was then transferred to 150,000 293T-ACE2-GFP cells and incubated on ice for 90 min. Following this, the cells were washed 2x with PBS followed by staining for surelight® APC conjugated anti-his antibody (Abcam, ab72579) for 30 min on ice. As a positive control, spike protein was pre-incubated with recombinant human ACE2 before transferring to 293T-ACE2-GFP cells. Data was acquired using a BD LSRII and analyzed by FlowJo (version 10).

Pseudovirus neutralization assay

SARS-CoV-2 pseudovirus was produced using HEK293T cells transfected with GeneJammer (Agilent) using IgE-SARS-CoV-2 spike plasmid (Genscript) and pNL4-3.Luc.R-E- plasmid (NIH AIDS reagent) at a 1:1 ratio. Forty-eight hours post transfection, supernatant was collected, enriched with FBS to 12% final volume, steri-filtered (Millipore Sigma), and aliquoted for storage at −80°C. SARS-Cov-2 pseudovirus neutralization assay was set up using D10 media (DMEM supplemented with 10%FBS and 1X Penicillin-Streptomycin) in a 96 well format. CHO cells stably expressing ACE2 were used as target cells (Creative Biolabs, Catalog No. VCeL-Wyb019). SARS-Cov-2 pseudovirus were titered to yield greater than 20 times the cells only control relative luminescence units (RLU) after 72h of infection. 10,000 CHO-ACE2 cells/well were plated in 96-well plates in 100ul D10 media and rested overnight at 37°C and 5% CO2 for 24 h. The following day, sera from INO-4800 vaccinated and control groups were heat inactivated and serially diluted. Sera were incubated with a fixed amount of SARS-Cov-2 pseudovirus for 90 min at RT. The sera+virus mix was then added to the plated CHO-ACE2 cells and allowed to incubate in a standard incubator (37% humidity, 5% CO2) for 72h. Cells were then lysed using Britelite plus luminescence reporter gene assay system (Perkin Elmer Catalog no. 6066769) and RLU were measured using the Biotek plate reader. Neutralization titers (ID50) were calculated using GraphPad Prism 8 and defined as the reciprocal serum dilution at which RLU were reduced by 50% compared to RLU in virus control wells after subtraction of background RLU in cell control wells.

Plaque reduction neutralization test

Live virus neutralization assays were performed by PRNT at Bioqual (Rockville, MD), on sera samples collected at pre-challenge (Weeks 0, 6, 15) and post-challenge. Vero 76 cells (ATCC No. CRL-1586) were plated at 175,000 cells/well in DMEM + 10% FBS + gentamicin, overnight at 37°C, 5% CO_2. Serially diluted serum samples were mixed with SARS-CoV-2 virus (isolate USA-WA1/2020) for a final 30 pfu/well virus per well and final starting 1:20 serum dilution, then incubated at 37°C, 5% CO_2 for 1 h. Media

was removed from cell cultures and virus-serum dilution mixtures were added to cells in duplicate and then incubated at 37°C, 5% CO_2 for 1 h for virus infection. After incubation, 0.5% methylcellulose media (Sigma #M0512-100G) was added to each well and plates were incubated at 37°C, 5% CO_2 for 3 days. Each well was then washed and fixed with methanol, then stained with 0.2% crystal violet (Sigma #HT901-8FOZ) in 20% MeOH for 30 min at room temperature. Plates were washed, dried and plaques were recorded to calculate the number of infectious units.

Antibody-dependent cellular phagocytosis

Biotinylated SARS-CoV-2 (COVID-19) S1 and RBD proteins (Acro Biosystems) were combined with fluorescent NeutrAvidin beads (Life Technologies) overnight at 4°C. Excess, unconjugated antigens were removed by washing the beads twice with 0.1% PBS-BSA. Beads were washed with 1 mL buffer and spun at 14,000 x g for 2 min at room temperature. Antigen-coated beads were resuspended in a final volume of 1 mL in PBS-BSA. 10 μL beads were added into each well of a round-bottom 96-well culture plate after which 5 μl of heat-inactivated monkey plasma were added and incubated for 2 h at 37°C. A 200 μl suspension of THP-1 cells at 2.5×10^5 cells/ml were to each well, for a total of 5×10^4 THP-1 cells per well. After mixing, the cell-bead mixtures were incubated overnight at 37°C. The following day, 100 μL of supernatant from each well were removed and 100 μL of BD Cytofix were added to each well. Cells were analyzed by flow cytometry and data collected were analyzed in FlowJo software. The percentage of fluorescent / bead+ cells and the median fluorescence intensity of the phagocytic cells were computed to determine the phagoscore.

Antibody-dependent complement deposition

4×10^6 (ACE2)-CHO cells were pulsed with 20 μg biotinylated SARS-CoV-2 (COVID-19) S1 and RBD proteins (Acro Biosystems) for 30 min at 37°C. Excess, unbound antigens were removed by washing cells once with complete medium. Heat-inactivated monkey plasma (10 μl) were added to the antigen-pulsed cells and incubated for another 30 min at 37°C. Freshly resuspended lyophilized guinea pig complement (Cedarlane) diluted 1:20 with veronal buffer 0.1% gelatin with calcium and magnesium (Boston BioProducts) were added to the cells for 2 h at 37°C. Following a wash with 1X PBS, cells were assessed for complement deposition by staining with goat anti-guinea pig C3-FITC (MP biomedicals). After fixing, cells were analyzed by flow cytometry and ADCD are reported as MFI of FITC+ cells.

Viral RNA assay

RT-PCR assays were utilized to monitor viral loads, essentially as previously described.[56] Briefly, RNA was extracted using a QIAcube HT (QIAGEN,Germany) and the Cador pathogen HT kit from bronchoalveolar lavage (BAL) supernatant and nasal swabs. RNA was reverse transcribed using superscript VILO (Invitrogen) and ran in duplicate using the QuantStudio 6 and 7 Flex Real-Time PCR System (Applied Biosystems) according to manufacturer's specifications. Viral loads were calculated of viral RNA copies per mL or per swab and the assay sensitivity was 50 copies. The target for amplification was the SARS-CoV2 N (nucleocapsid) gene. The primers and probes for the targets were:

2019-nCoV_N1-F:5′-GACCCCAAAATCAGCGAAAT-3′
2019-nCoV_N1-R: 5′-TCTGGTTACTGCCAGTTGAATCTG-3′
2019-nCoV_N1-P: 5′-FAM-ACCCCGCATTACGTTTGGTGGACC-BHQ1-3′

Subgenomic mRNA assay

SARS-CoV-2 E gene subgenomic mRNA (sgmRNA) was assessed by RT-PCR using an approach similar to previously described.[41] To generate a standard curve, the SARS-CoV-2 E gene sgmRNA was cloned into a pcDNA3.1 expression plasmid; this insert was transcribed using an AmpliCap-Max T7 High Yield Message Maker Kit (Cellscript) to obtain RNA for standards. Prior to RT-PCR, samples collected from challenged animals or standards were reverse-transcribed using Superscript III VILO (Invitrogen) according to the manufacturer's instructions. A Taqman custom gene expression assay (ThermoFisher Scientific) was designed using the sequences targeting the E gene sgmRNA.[41] Reactions were carried out on a QuantStudio 6 and 7 Flex Real-Time PCR System (Applied Biosystems) according to the manufacturer's specifications. Standard curves were used to calculate sgmRNA in copies per ml or per swab; the quantitative assay sensitivity was 50 copies per ml or per swab.

QUANTIFICATION AND STATISTICAL ANALYSIS

Statistical analyses were performed using GraphPad Prism 8.0 software (La Jolla, CA). All bar graphs, scatterplots, and line graphs display individual animals or the mean value, and error bars represent the standard deviation. A two-tailed Mann-Whitney test was performed for viral load comparison between vaccinated and unvaccinated animals. Samples and animal groups with a p value < 0.05 were considered statistically significant. Statistical details can be found in the Results, figure legends, and on the figures.

Cell Reports Medicine, Volume *2*

Supplemental information

Intradermal-delivered DNA vaccine induces durable immunity mediating a reduction in viral load in a rhesus macaque SARS-CoV-2 challenge model

Ami Patel, Jewell N. Walters, Emma L. Reuschel, Katherine Schultheis, Elizabeth Parzych, Ebony N. Gary, Igor Maricic, Mansi Purwar, Zeena Eblimit, Susanne N. Walker, Diana Guimet, Pratik Bhojnagarwala, Opeyemi S. Adeniji, Arthur Doan, Ziyang Xu, Dustin Elwood, Sophia M. Reeder, Laurent Pessaint, Kevin Y. Kim, Anthony Cook, Neethu Chokkalingam, Brad Finneyfrock, Edgar Tello-Ruiz, Alan Dodson, Jihae Choi, Alison Generotti, John Harrison, Nicholas J. Tursi, Viviane M. Andrade, Yaya Dia, Faraz I. Zaidi, Hanne Andersen, Mohamed Abdel-Mohsen, Mark G. Lewis, Kar Muthumani, J. Joseph Kim, Daniel W. Kulp, Laurent M. Humeau, Stephanie J. Ramos, Trevor R.F. Smith, David B. Weiner, and Kate E. Broderick

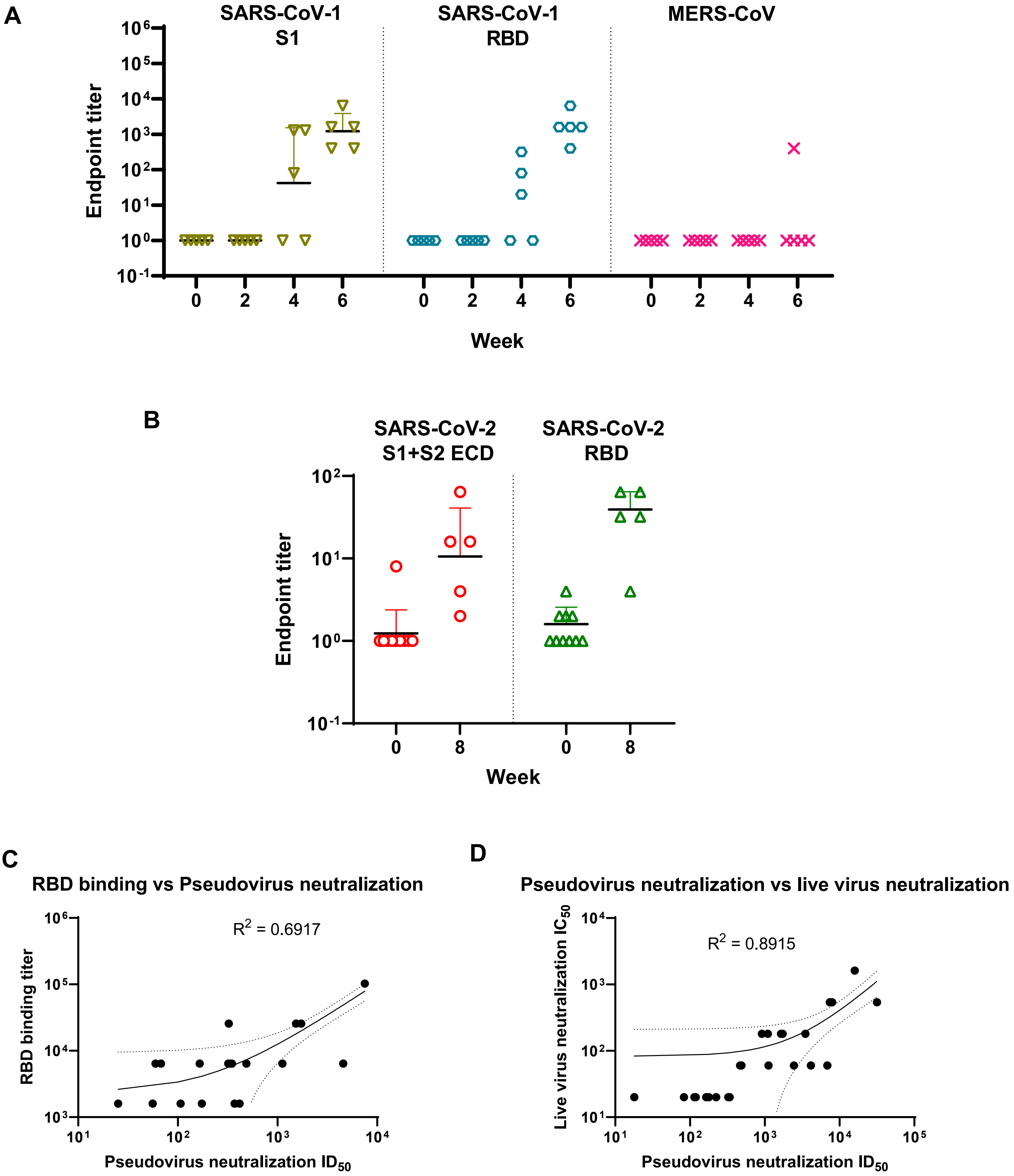

Figure S1. Immune Responses in INO-4800 vaccinated rhesus macaques, Related to Figure 1 **A)** Serum IgG cross-reactivity to SARS-CoV and MERS-CoV spike protein. IgG binding was measured in sera from INO-4800 vaccinated rhesus macaques to SARS-CoV S1 and MERS-CoV S1 protein antigen. **B)** Bronchoalveolar lavage (BAL) IgG reactive to SARS-CoV-2 S protein antigens. BAL samples collected from vaccinated animals were assessed for SARS-CoV-2 reactive IgG binding to the full-length SARS-CoV-2 spike protein and the RBD domain. **C and D)** Simple linear regression analysis comparing total IgG antibodies against (**C**) the RBD and (**D**) neutralizing antibodies raised in DNA-vaccinated macaques

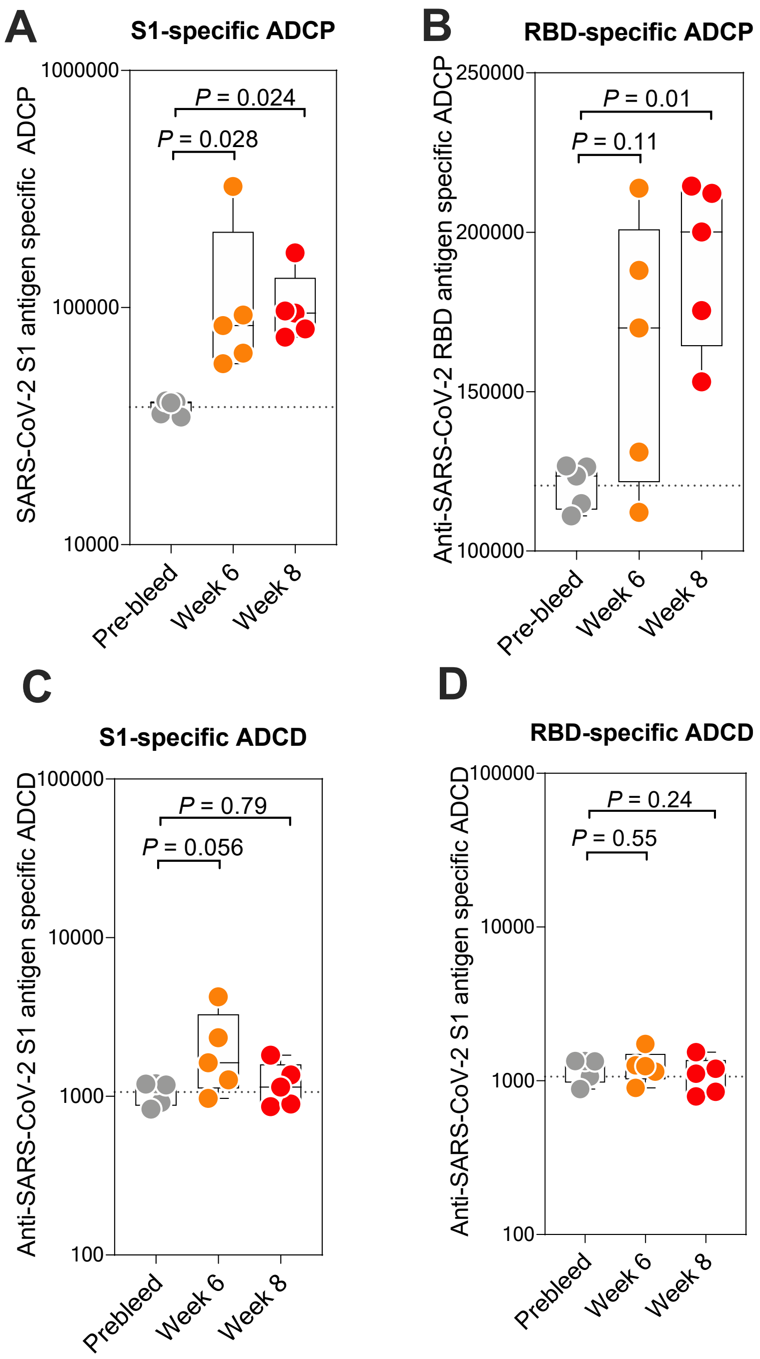

Figure S2. Antibody-dependent cellular phagocytosis (ADCP) and antibody-dependent complement deposition (ADCD) activity of NHP sera at peak immune response time points post-vaccination, Related to Figure 1. To measure ADCP activity, sera from immunized macaques were combined with SARS-CoV-2 A) S1 or B) RBD-conjugated fluorescent beads, followed by overnight incubation with THP-1 cells. The phagoscore was determined by calculating the percentage of fluorescent / bead+ cells and the median fluorescence intensity of the THP-1 cells. To measure ADCD activity, ACE2-CHO cells were pulsed with biotinylated SARS-CoV-2 C) S1 and D) RBD proteins and subsequently combined with NHP sera. Freshly diluted guinea pig complement was added to the cells and following incubation, complement deposition was detected with a goat anti-guinea pig C3-FITC conjugated antibody and analyzed by flow cytometry. ADCD is reported as MFI of FITC+ cells.

A

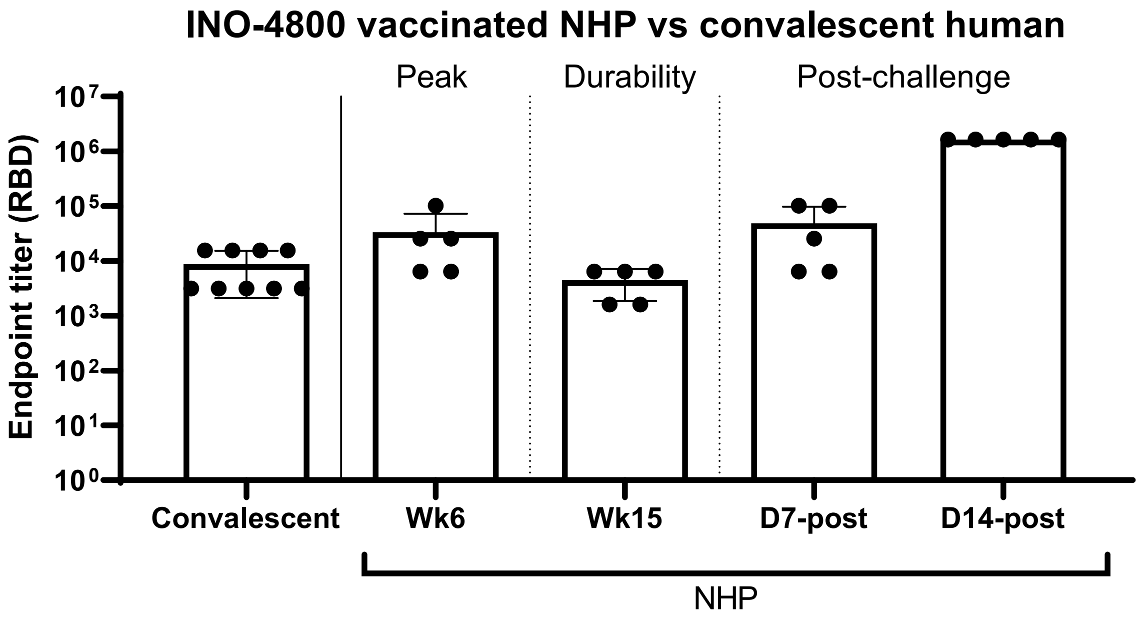

B

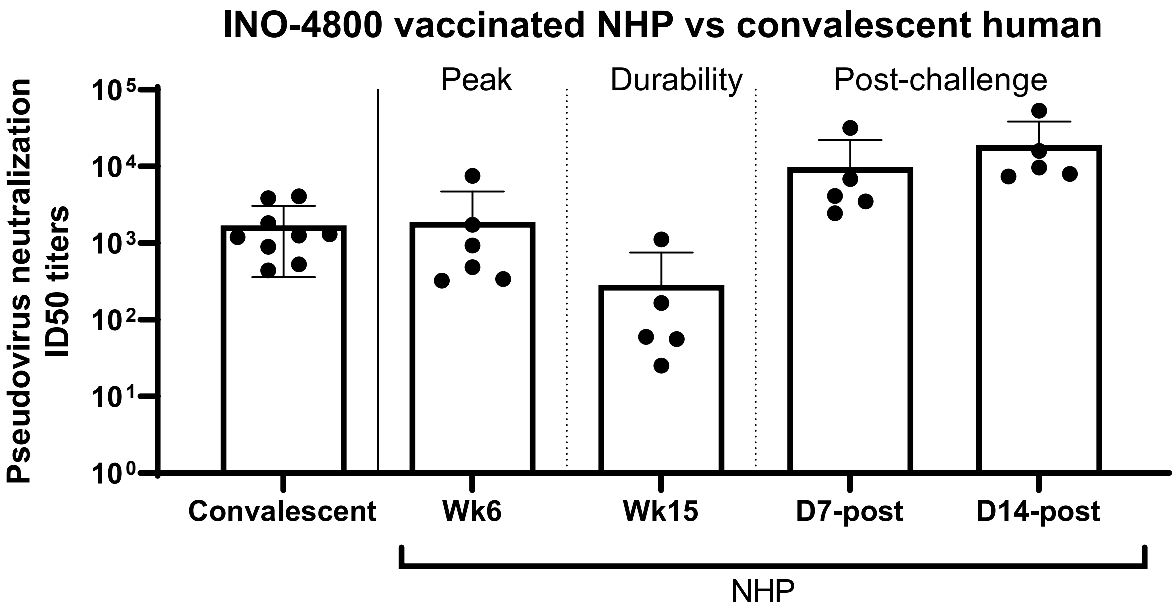

Figure S3. Comparison of NHP endpoint binding antibody and pseudoneutralization titers with SARS-CoV-2 convalescent human donors, Related to Figures 1 and 3. (**A**) ELISA endpoint titer comparison (RBD) and (**B**) Pseudoneutralization assay (D614) comparing INO-4800 vaccinated NHP peak, durability, and post-challenge pseudoneutralization ID50 titers with SARS-CoV-2 convalescent human sera titers.

A

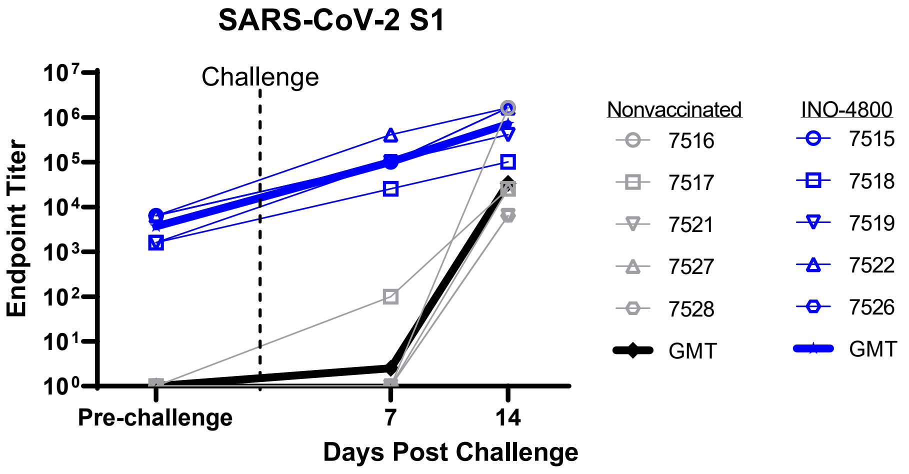

B

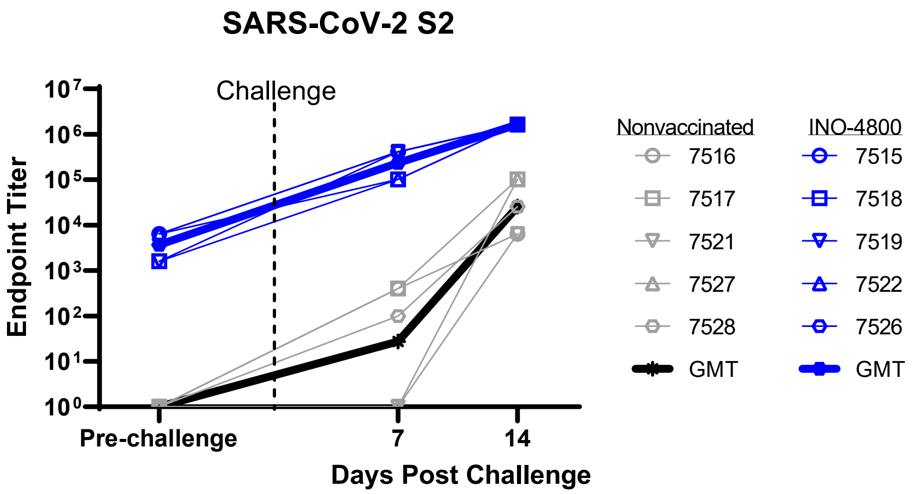

Figure S4. **Recall of humoral immune responses after viral challenge, Related to Figure 3.** (**A**) SARS-CoV-2 S1 protein and (**B**) SARS-CoV-2 S2 protein antigen binding of IgG in diluted NHP sera collected prior to challenge, during challenge and post challenge.
